# Chemical exposomics in biobanked plasma samples and associations with breast cancer risk factors

**DOI:** 10.1038/s41370-024-00736-0

**Published:** 2024-12-06

**Authors:** Jessica Edlund, Kalliroi Sdougkou, Stefano Papazian, Wendy Yi-Ying Wu, Jonathan W. Martin, Sophia Harlid

**Affiliations:** 1 Department of Diagnostics and Intervention, Oncology, Umeå University, Umeå, 901 87 Sweden; 2 Department of Environmental Science, Science for Life Laboratory, Stockholm University, Stockholm, 106 91 Sweden; 3 National Facility for Exposomics, Metabolomics Platform, Science for Life Laboratory, Stockholm University, Solna, 171 65 Sweden

**Keywords:** Chemical exposome, Breast cancer, High-resolution mass spectrometry, Liquid chromatography, Plasma

## Abstract

**Background:**

The chemical exposome includes exposure to numerous environmental and endogenous molecules, many of which have been linked to reproductive outcomes due to their endocrine-disrupting properties. As several breast cancer risk factors, including age and parity, are related to reproduction, it is imperative to investigate the interplay between such factors and the chemical exposome prior to conducting large scale exposome-based breast cancer studies.

**Objective:**

This pilot study aimed to provide an overview of the chemical exposome in plasma samples from healthy women and identify associations between environmental exposures and three risk factors for breast cancer: age, parity, and age at menarche.

**Material and methods:**

Plasma samples (*n* = 161), were selected based on reproductive history from 100 women participating in the Northern Sweden Health and Disease Study, between 1987 and 2006. Samples were analyzed by liquid chromatography high-resolution mass spectrometry (LC-HRMS) for 77 priority target analytes including contaminants and hormones, with simultaneous untargeted profiling of the chemical exposome and metabolome. Linear mixed effects models were applied to test associations between risk factors and chemical levels.

**Results:**

Fifty-five target analytes were detected in at least one individual and over 94,000 untargeted features were detected across all samples. Among untargeted features, 430 could be annotated and were broadly classified as environmental (246), endogenous (167) or ambiguous (17). Applying mixed effect models to features detected in at least 70% of the samples (16,778), we found seven targeted analytes (including caffeine and various per- and poly-fluoroalkyl substances) and 38 untargeted features, positively associated with age. The directionality of these associations reversed for parity, decreasing with increasing births. Seven separate targeted analytes were associated with age at menarche.

**Significance:**

This study demonstrates how a comprehensive chemical exposome approach can be used to inform future research prioritization regarding associations between known and unknown substances, reproduction, and breast cancer risk.

**Impact statement:**

- This study illustrates how chemical exposomics of long-term stored blood samples offers valuable insights to discover chemical exposures and their potential links to disease in humans, particularly those related to reproduction and breast cancer risk factors.

## Introduction

Breast cancer is a hormone sensitive disease with possible links to endocrine disrupting chemical (EDC) exposure [1–3]. Legacy and current use EDCs include various pesticides, parabens, and perfluoroalkyl substances (PFAS), of which several have been previously banned or restricted due to their toxicity and associations with disease and reproductive health [4, 5]. EDCs may interact with cellular receptors or plasma proteins to interfere with hormonal homeostasis, and early life exposure may also result in long-lasting adverse effects on mammary gland development [6].

As breast cancer risk is strongly associated with a woman’s lifetime estrogen exposure, EDCs that mimic hormonal effects may also be indirectly related to breast cancer risk, though e.g. effect modification [7], as their concentrations may be associated with a woman’s age, parity, age at menarche or menopause. Identifying how chemical exposure intensity varies in relation to reproductive factors can therefore be of high importance for disentangling environmental contributions to cancer and reproductive risks, while also leading to better understanding of environmental chemical disposition during development, pregnancy, and aging.

Examples of the latter include serum concentrations of various PFAS, which are generally lower in parous compared to non-parous women [8], while perfluoroundecanoate (PFUnDA), perfluorododecanoate (PFDoDA) and perfluorooctanoate (PFOA) are associated with maternal age [9]. Higher levels of PFAS in blood have also been associated with an earlier onset of menarche [10], although evidence is inconsistent [11, 12].

The human exposome encompasses the cumulative sum of environmental exposures over an individual’s lifetime and includes exposures to chemicals from external and internal sources such as pollution, drugs, diet, and the metabolic products of the gut microbiota [13, 14]. Measuring the chemical exposome in blood presents multiple advantages, including the potential to simultaneously measure other small-molecule endogenous metabolites that may be impacted by external exposures, e.g. reproductive hormones [15].

Despite increasing evidence for associations between environmental exposures and breast cancer development and etiology, most previous studies have only assessed single chemicals or metabolites, or well-defined chemical groups when evaluating such associations [1, 2]. With hundreds of thousands of chemicals in commerce and a range of chemical classes already implicated in the development of chronic diseases, including cancer [16], more comprehensive chemical exposomics approaches are increasingly applied to identify harmful exposures [17]. Sensitive multiclass target and untargeted methods based on liquid chromatography (LC) and high-resolution mass spectrometry (HRMS) have been demonstrated for the simultaneous profiling of environmental and endogenous substances in blood plasma [17–19].

We hypothesized that the chemical burden would increase as women age, for most chemicals, but that parity (especially number of deliveries) would be associated with lower levels. For age at menarche, we hypothesized that the level of chemical exposure would be higher in women with earlier menarche onset as individuals might start using hygiene products at an earlier age. In this pilot study we explored the chemical exposome in 161 plasma samples from 100 Swedish women to identify possible associations between chemical exposures and breast cancer reproductive risk factors, specifically age at sampling, parity (number of pregnancies and deliveries) and age at menarche. Moreover, longitudinal exposures were evaluated using linear mixed models by incorporating data from a subgroup of participants (*n* = 61) who provided a second plasma sample up to 16 years after the first visit.

## Materials and methods

### Study population

Plasma samples (*n* = 161) originated from participants of the Northern Sweden Health and Disease Study (NSHDS) [20]. All NSHDS blood samples are collected in EDTA and heparin tubes, and aliquots of plasma, buffy coat and erythrocytes are stored at −80 °C at the Northern Sweden Biobank (Biobanken Norr) in Umeå, Sweden. NSHDS consists of three sub-cohorts: the Västerbotten Intervention Programme (VIP); the Multinational Monitoring of Trends and Determinants in Cardiovascular Disease (MONICA) study; and the Mammography Screening Project (MSP). Of these, the VIP is still ongoing, and MONICA completed their latest recruitment in 2023. The MSP was initiated in 1995, and recruited women in the Swedish county of Västerbotten during routine mammography screening. Participants donated a blood sample upon enrollment and answered a short questionnaire focused on reproductive factors such as number of previous pregnancies and deliveries, age at menarche and menopause, as well as previous use of hormonal contraception or menopausal hormone therapy. Recruitment to MSP ended in 2006.

The current study included 100 women, free from breast cancer, that had donated at least one sample to the MSP. Women were selected based on reproductive history, with 26% reporting no previous pregnancy, 23% reporting 1-2 pregnancies, 27% reporting 3-4 pregnancies and 24% reporting more than 4 pregnancies. As a selection criterion for the study, women were included if reporting at least one pregnancy (*n* = 74) with a blood sample collected, at the latest, within 2 years from their last pregnancy. A subset of the of the participants (*n* = 61; 82%) also provided a second sample up to 16 years after the first clinical visit. Besides number of pregnancies, additional metadata available for the selected individuals included the number of deliveries, age at the sampling occasions, body mass index (BMI), age at menarche and menopause, use of contraceptive pills, use of hormone replacement therapy, smoking, and snuff status. The number of pregnancies comprised all reported pregnancies (including those terminated early such as miscarriages), whereas number of deliveries referred to all reported births.

### Plasma sample preparation

Heparin plasma samples were prepared by a phospholipid depletion technique and analyzed by a combined targeted and untargeted chemical exposomics method described by Sdougkou et al. [19]. Briefly, 200 µL plasma aliquots were placed in 2 mL tubes and fortified with 10 µL of an isotope-labeled internal standard mixture (34 substances) in methanol (MeOH) (Optima LC/MS Grade, Thermo Scientific) (Supplementary Table S1, final concentration 1 ng/mL of each). Protein precipitation was achieved by adding 800 µL acetonitrile (ACN, Optima LC/MS Grade, Fisher Chemical) containing 0.5% citric acid (BioUltra, 112 anhydrous, ≥99.5%). Samples were vortexed for 20 s, left to rest at 4 °C for 20 min, and then centrifuged (20,800 × *g*) at 4 °C for 10 min. Supernatants were loaded onto HybridSPE-Phospholipid cartridges (500 mg/6 mL, Merck) that had been preconditioned with 12 mL MeOH and 12 mL ACN containing 0.5% citric acid. Elution was performed with 1 mL ACN containing 0.5% citric acid, followed by 2 mL MeOH containing 1% ammonium formate (LiChropur, ≥99.0%) and collected into 15 mL tubes. The pH of the extracts was adjusted from approximately pH 3 to 6.5 by adding 40 µL of 25% ammonia solution (LiChropur) and centrifuging for 10 min at 4300 × *g*. Supernatants were transferred to 5 mL tubes, evaporated to 100 µL under nitrogen flow and subsequently ultrasonicated for 5 min. A final rinse of the tubes with 100 µL MeOH was performed to reach a final extract volume of 200 µL, followed by centrifugal filtration (10,600 × *g*) for 10 min (0.2 μm nylon filters, Thermo Scientific). The final extracts were transferred to amber glass vials and spiked with 10 μL of the internal standard diuron-d6 solution (final concentration 4 ng/mL) to correct for extract volume variations and to monitor instrumental performance.

### LC-HRMS analysis

Parallel targeted and untargeted analysis was performed by ultrahigh pressure LC (Ultimate 3000, Thermo Scientific) with HRMS acquisition (Q Exactive Orbitrap HF-X, Thermo Scientific) in positive and negative electrospray ionization mode (ESI+ and ESI-) as previously described [19]. Spectral acquisition alternated between full scan (i.e. MS1; 90-1000 mass-to-charge ratio (*m/z*), 120,000 nominal resolution) and data-independent acquisition (DIA) MS/MS (i.e. MS2; 30,000 nominal resolution, product ion scan range starting from 50 Da) with four *m/z* precursor windows of equal size (237 Da) which overlapped by 10 Da. Injection volumes were 20 μL, corresponding to 20 µL plasma-equivalents on-column, with chromatography performed at 40 °C on an Acquity BEH C18 column (130 Å, 1.7 μm, 3 ×100 mm, Waters) with an Acquity BEH C18 1.7 µM vanguard pre-column. Upstream of the injector, an Acquity BEH C18 column (130 Å, 1.7 μm, 3 ×30 mm, Waters) was placed to separate instrumental background analytes from sample analytes. A binary gradient elution at 0.4 mL/min used mobile phases (A) water (Optima LC/MS Grade, Thermo Scientific) containing 1 mM ammonium fluoride (Honeywell Fluka, ≥98.0%), and (B) 100% MeOH. The elution gradient started at 5% B, linearly increased to 100% B by 15 min (held until 22 min) and returned to initial conditions with 4 min equilibration.

### Target analyte quantification

The chemical exposomics method was quantitatively validated for 77 targeted analytes, including environmental contaminants, dietary chemicals, tobacco markers, drugs and endogenous steroid hormones [19]. A complete list of target analytes and their corresponding method limits of quantification (MLOQ) is presented in Supplementary Table S2. Four precursors of perfluorooctane sulfonate (PFOS), i.e. perfluorooctane sulfonamidoacetic acid (FOSAA), its *N*-methyl- and *N*-ethyl- derivatives (NMeFOSAA and NEtFOSAA), and *N*-methyl perfluorobutane sulfonamide (NMeFBSA) were added after preliminary method validation (instrumental LOQ reported in Supplementary Table S2). Peak areas of the target analytes were integrated in Xcalibur Quan Browser (Thermo Scientific, v.4.1) using solvent-based calibration curves with internal standards (9 points, 0.01–100 ng/mL) for quantification. The calibration range was selected to fit the expected relatively low concentrations of environmental contaminants in blood plasma. However, concentrations higher than the upper calibration range were measured for six of the quantified analytes, i.e. diclofenac, ibuprofen, paracetamol, cotinine, caffeine and hydrocortisone (in 1%, 2%, 10%, 18%, 75% and 76% of the samples respectively). In these cases, linear extrapolation was performed to calculate concentrations. Branched PFAS isomers, which were chromatographically resolved from the corresponding linear isomers, were quantified separately. Reference standardization [21] was used to quantify targeted steroid hormones.

The 161 plasma samples were extracted and analyzed in five injection batches. Within each batch, calibration curves were injected 3 times (beginning, middle, end of the sequence) to account for any instrumental drift. A pooled plasma sample, also from the NSHDS cohort, was run multiple times within each injection sequence to support reference standardization and enable future retrospective semi-quantification for newly discovered untargeted analytes. For data summaries and statistics, when analytes were detected at concentrations lower than the respective MLOQ, the analyte concentrations were substituted by MLOQ/2, while for non-detect analytes concentrations were substituted by MLOQ/4.

### Untargeted data pre-processing, data treatment, and chemical annotations

For untargeted analysis, raw data were pre-processed in MS-DIAL (v.4.92) [22], allowing feature alignment across samples, MS1 and DIA MS2 spectral deconvolution, and peak area integration (Detailed MS-DIAL processing parameters are reported in Supplementary Table S3). Each molecular feature was defined by a chromatographic retention time (RT), peak area, accurate mass MS1 (*m/z*) and a deconvoluted MS2 spectrum. Only those features with peak areas at least 5 times higher than the corresponding procedural blanks (*n* = 2 blanks per batch) were retained (i.e., sample maximum/blank average >5).

Downstream data treatment was performed in Python (v. 3.8.16) using Jupyter Notebook (v. 6.5.2). Putative redundant features detected in both ESI+ and ESI− were estimated using a RT tolerance of 0.2 min, and an *m/z* tolerance of 0.002 Da based on calculated M from [M − H]− in negative mode and [M + H]+ in positive mode. In the case of ESI+/− dataset overlap, the feature with the lowest signal intensity (average peak area) was discarded in the corresponding ESI mode. The combined ESI+/− dataset was normalized to correct for MS instrumental variation using the multivariate principal component analysis (PCA) scores of isotope-labeled internal standard signal intensities in each sample [23, 24].

In further data treatment, the ratio of each sample’s peak area to the average procedural blank peak area was computed for each feature; 0.1 was added to the average procedural blank area of each feature to avoid 0 in the denominator when there was no blank response. For each feature, samples with peak areas lower than 5 times the corresponding blank average were set to 0. Average areas in blank (when detectable) were subtracted from peak areas in samples which passed the threshold. This additional blank filtering / subtraction step constrained potential artefacts in the final dataset that would lead to a misleading higher detection frequency (DF). At this stage, peaks with areas below 90,000 were set to 0. As a final data reduction step, highly correlating annotated features (Pearson correlation coefficient (r) >0.95, *p*-value < 0.001) with RTs differing by a maximum of 0.1 min, and with identical peak shapes (assessed by visual inspection) were considered as likely originating from the same analyte (e.g. produced by in-source fragmentation or adduct); the feature with lowest average peak area was then discarded.

For chemical annotations at confidence Level 2 [25], spectral library matching were considered when the total identification score in MS-DIAL was >700 (i.e., corresponding to dot- or reverse dot-product scores >600) using open databases at MassBank Europe (https://massbank.eu/), MassBank of North America (https://mona.fiehnlab.ucdavis.edu/) and Global Natural Product Social Molecular Networking (GNPS; https://gnps.ucsd.edu/). Confidence Level 1 [25] was assigned when annotation was performed by matching the in-house spectral library of reference standards with RT information on the same LC system. For each annotated feature, a class (i.e. endogenous or environmental) and subclass (e.g. bile acids, PFAS) was assigned by integrating metadata from PubChem [26], the Human Metabolome Database (HMDB) [27], and the Food Metabolome Database (FooDB) [28].

### Statistical analysis

All features with a detection frequency lower than 70% were excluded from statistical analysis, and target analytes were also excluded if the combined detection frequency (i.e. MLOQ/4 and MLOQ/2) was lower than 60%. Concentrations of linear and branched PFAS isomers (when detected) were summed as total concentrations, e.g. total PFOS. After excluding analytes with high numbers of non-detect, 18 targeted analytes were retained in the following data analysis. We also retained cotinine and progesterone due to interest, despite both falling slightly below the cut-off criteria for target analytes. The distribution of the remaining target chemicals was visualized (Supplementary Fig. S1A) and transformed by raising to power 1/2 to adjust for left skewed distributions and approximate normality (Supplementary Fig. S1B). Time trends were visualized between the first and second sample for the 61 women providing two samples.

Correlations between all non-transformed chemical concentrations and the reproductive risk factors were analyzed using Spearman’s correlation test. For substances belonging to the same chemical class, we further assessed correlations between transformed concentrations using Pearson´s correlation tests and combined correlated chemicals into three groups based on visual inspection of clustered chemicals in the correlation matrix (Supplementary Fig. S2). The transformed values for all chemicals belonging to the same group were then summed up and their combined value used as the outcome variable in downstream analyses.

Linear mixed effect models (lmerTest package in R) with subject-specific random intercepts were applied to assess the influence of the breast cancer risk factors on the chemical levels. Each chemical was modeled as a separate outcome and the reproductive risk factors tested included age, number of pregnancies, number of deliveries, and age at menarche. Potential interaction between age and number of pregnancies was assessed by including an interaction term in the model that included both age and number of pregnancies. All analyses were adjusted for appropriate confounders as was determined by directed acyclic graphs [29] as well as individually assessed relationships between exposures and outcomes. We also performed a sensitivity analysis, excluding all nulliparous women, when testing associations between age and chemical burden. For analyses involving the targeted chemicals an alpha of 0.05 was used as the threshold for significance whereas in analyses of untargeted features the significance levels were adjusted for multiple testing using a *p*-value threshold of 0.005 as proposed by Benjamin et al. [30] and a Bonferroni-level cutoff. All statistical analyses were carried out in R (v. 4.0.3) and R Studio (v. 1.2.5019). For circular data visualization the R package circlize (v.0.4.15) was used [31].

## Results

A summary of participant characteristics is presented in Table 1. The 100 women included in the study had a mean age of 43.4 years at their first sampling. Nulliparous women (who only provided one sample for this study) where considerably older with a mean age of 58.3 years, compared to 38.4 years at the first and 45.7 years at the second sampling occasion for parous women. Nulliparous women were also born almost two decades prior to parous women (average birth year was 1941 and 1958, respectively). The age at menarche (average 13 years old) was similar for all women regardless of number of pregnancies reported.

**Table 1:** Participant characteristics and concentrations of targeted analytes.

Variable	Nullipara (*n* = 26)	1-2 pregnancies^a^ (*n* = 23)	3-4 pregnancies^a^ (*n* = 27)	>4 pregnancies^a^ (*n* = 24)
Provided a second blood sample, *n*	0	22	21	18
Age at first sample, *mean (SD)*	58.28 (6.48)	34.77 (5.33)	39.99 (4.72)	40.36 (3.09)
Age at second sample, *mean (SD)*	N/A	43.58 (4.13)	46.35 (3.98)	47.16 (4.83)
First sample collected, *median (range)*	1998 (1995, 2006)	1992 (1987, 2005)	1996 (1988, 2006)	1998 (1988, 2002)
Second sample collected, *median (range)*	N/A	2002 (1997, 2006)	2002 (2000, 2006)	2002 (1996, 2006)
Birth year, *median (range)*	1941 (1927, 1955)	1960 (1947–1975)	1956 (1948–1964)	1957 (1946–1961)
Number of deliveries^b,c^*, n (%)*				
No delivery	26 (100.00)	0 (0.00)	1 (3.70)	0 (0.00)
1–2 deliveries	0 (0.00)	23 (100.00)	7 (25.93)	6 (25.00)
3–4 deliveries	0 (0.00)	0 (0.00)	19 (70.37)	12 (50.00)
>4 deliveries	0 (0.00)	0 (0.00)	0 (0.00)	6 (25.00)
Age at menarche, *mean (SD)*	13.45 (1.31)	12.56 (1.07)	13.04 (1.48)	12.92 (1.33)
Missing	1	0	0	0
Body Mass Index^c^, *mean (SD)*	25.36 (4.03)	23.07 (2.91)	25.02 (5.38)	24.59 (3.75)
18-25, *n (%)*	11 (42.31)	17 (73.91)	17 (62.96)	17 (70.83)
>25, *n (%)*	15 (57.69)	6 (26.09)	10 (37.04)	7 (29.17)
Ever used tobacco^c,d^, *n (%)*				
Yes	4 (15.38)	12 (52.17)	11 (40.74)	11 (45.83)
No	22 (84.62)	11 (47.83)	16 (59.26)	13 (54.17)
Ever use hormone therapy^e^, *n (%)*				
Yes	4 (15.38)	3 (13.04)	2 (7.41)	3 (12.50)
No	19 (73.08)	15 (65.22)	23 (85.19)	21 (87.50)
Missing	3 (11.54)	5 (21.74)	2 (7.41)	0 (0.00)
Ever use hormonal birth control^c^, *n (%)*				
Yes	11 (42.31)	22 (95.65)	21 (77.78)	21 (87.50)
No	15 (57.69)	1 (4.35)	4 (14.81)	1 (4.17)
Missing	0 (0.00)	0 (0.00)	2 (7.41)	2 (8.33)
Target analyte concentrations (ng/mL)^c^, *mean (SD)*				
Corticosterone	5.04 (4.16)	7.37 (7.99)	6.66 (4.37)	5.65 (3.30)
Hydrocortisone	127.83 (51.42)	161.95 (68.43)	147.59 (55.26)	140.58 (38.01)
Progesterone	0.48 (2.32)	4.12 (6.64)	3.11 (6.08)	3.21 (5.58)
Testosterone	0.17 (0.07)	0.18 (0.07)	0.18 (0.10)	0.17 (0.08)
Pentachlorophenol	1.24 (1.11)	2.10 (1.79)	1.92 (1.97)	1.57 (1.36)
Caffeine	419.40 (491.40)	94.94 (92.40)	185.52 (179.09)	187.08 (165.42)
Cotinine	47.94 (100.76)	58.87 (125.75)	23.32 (70.82)	63.44 (155.04)
TCPy	0.24 (0.30)	0.30 (0.23)	0.31 (0.39)	0.52 (0.72)
PFHpA	0.07 (0.11)	0.04 (0.04)	0.04 (0.03)	0.05 (0.04)
NMeFOSAA	0.64 (0.67)	0.32 (0.19)	0.32 (0.23)	0.47 (0.28)
PFHxS (total)	1.52 (1.10)	0.58 (0.32)	0.56 (0.22)	0.61 (0.19)
PFOA (total)	3.43 (1.70)	1.62 (0.59)	1.59 (0.74)	1.63 (0.61)
PFHpS (total)	0.45 (0.46)	0.19 (0.06)	0.19 (0.08)	0.19 (0.06)
PFOS (total)	23.40 (18.25)	15.11 (6.21)	12.47 (5.16)	12.99 (3.92)
NEtFOSAA	1.21 (2.50)	1.49 (1.88)	0.96 (1.23)	0.96 (0.71)
FOSAA	0.76 (0.98)	1.56 (2.04)	0.83 (0.70)	0.91 (0.69)
FOSA (total)	0.36 (0.30)	0.58 (0.63)	0.34 (0.26)	0.33 (0.19)
PFUnDA	0.22 (0.08)	0.21 (0.08)	0.18 (0.07)	0.17 (0.08)
PFNA	0.73 (0.29)	0.35 (0.13)	0.38 (0.13)	0.39 (0.17)
PFDA	0.23 (0.09)	0.15 (0.06)	0.16 (0.05)	0.15 (0.05)

^a^Number of pregnancies refers to reported pregnancies and includes pregnancies terminating early, e.g., miscarriages.

^b^Number of deliveries refers to reported deliveries.

^c^At the first sampling occasion.

^d^Includes both smoking and snuff use.

^e^At the last sampling occasion.

Tobacco use was lower among nulliparous women (approximately 15% reported previous tobacco use compared to 46% among parous women), as was previous use of hormonal contraception (42% among nulliparous and 77–96% among parous women).

### Target exposure analysis and associations with breast cancer reproductive risk factors

**Fig. 1:**
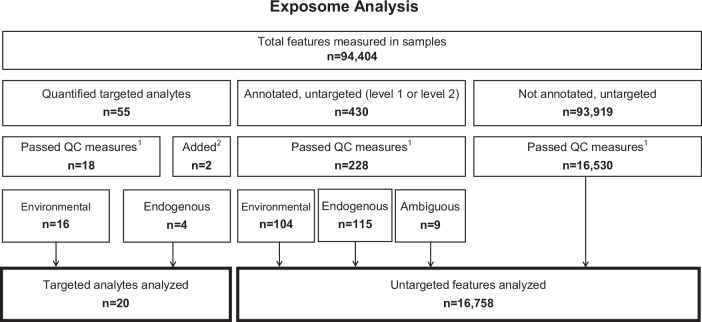
Flow chart showing selection of targeted analytes and untargeted features included in downstream analyses of associations with reproductive breast cancer risk factors. ^1^Measurable in more than 70% of samples. ^2^Added due to interest and close to QC-threshold.

In all plasma samples, 55 targeted analytes were detected at least once, belonging to 13 different chemical classes, including PFAS, flame retardants, phthalate metabolites, pharmaceuticals, substances from personal care products, pesticides and their metabolites, natural and artificial dietary substances, and tobacco biomarkers (Fig. 1, Supplementary Table S4). The endogenous hormones progesterone, testosterone, hydrocortisone, and corticosterone were also quantifiable in the majority of samples (Fig. 1, Supplementary Table S4). This total also included the branched isomers of PFOS, PFHxS, perfluoroheptane sulfonate (PFHpS), perfluorooctanoate (PFOA) and perfluorooctane sulfonamide (FOSA), which were quantified separately from their respective linear isomers but were summed for statistical analysis. The 20 target analytes that remained after excluding those detected in <70% of samples belonged to six different chemical classes, including food related chemicals, fungicides, insecticides, PFAS, steroid hormones and tobacco chemicals (Fig. 1, Supplementary Table S4). Some environmental chemicals were detected at higher concentrations among the nulliparous compared to parous women (e.g., caffeine, perfluorononanoate (PFNA), perfluorohexane sulfonate (PFHxS) and PFOS), while others, such as pentachlorophenol and perfluorooctane sulfonamido acetic acid (FOSAA) were higher among parous compared to nulliparous women (Table 1). Many of the PFAS analytes were also highly correlated (Supplementary Fig. S2) and thus were analyzed both separately and as clustered groups: i.e., Group 1: perfluorodecanoate (PFDA), PFNA, and PFUnDA; Group 2: FOSA, FOSAA and NEtFOSSA; and Group 3: PFOS, PFHpS, PFOA, and PFHxS.

**Fig. 2:**
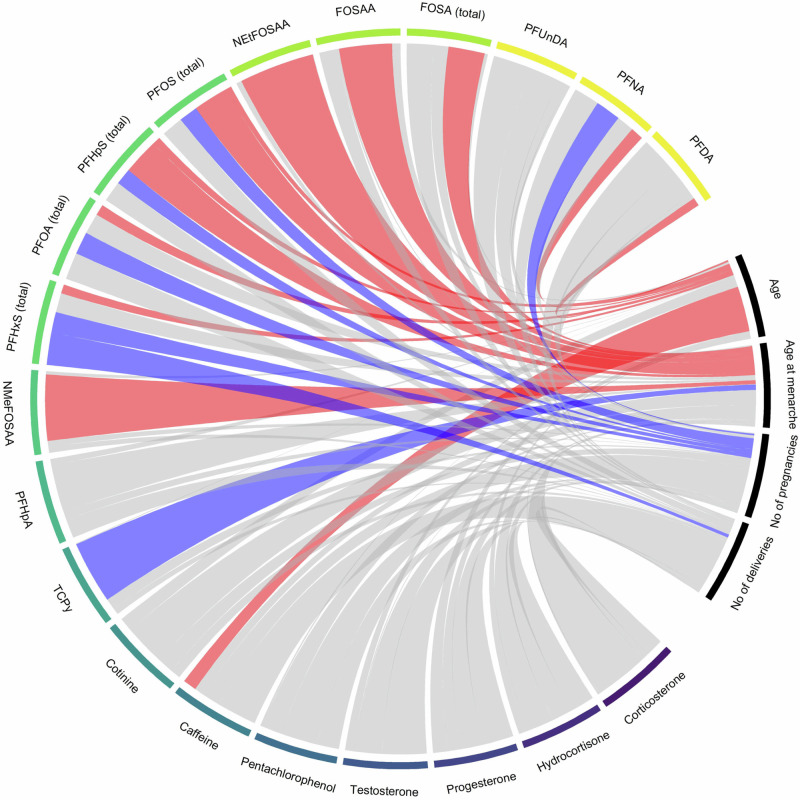
Circle diagram showing significant (*p* < 0.05) associations between different breast cancer risk factors and each target analyte (*n* = 20). Positive associations are depicted in red and negative associations in blue. Gray lines represent non-significant associations.

Several associations between individual target analytes and reproductive breast cancer risk factors were detected (Table 2) and were visualized (Fig. 2). Significant positive associations with age were found for seven analytes, i.e. caffeine, PFNA, PFHxS, PFOA, perfluoroheptanesulfonic acid (PFHpS), PFOS, and PFDA. One analyte (FOSAA) was negatively associated with age, but with borderline significance (*p*-value = 0.08). On the contrary, when analyzing associations with parity, we found that five PFAS analytes (PFHxS, PFOA, PFHpS, PFOS and PFNA) instead displayed negative coefficients, reaching significance for number of pregnancies, but generally not for number of deliveries (except for PFHxS). A positive association with age at menarche was found for six analytes (FOSAA, NMeFOSAA, NEtFOSAA, PFHpS, PFOS and FOSA), whereas a negative association was found for the herbicide metabolite, 3,5,6-trichloro-2-pyridinol (TCPy) (Fig. 2, Table 2).

**Table 2:** Associations between breast cancer risk factors and individual target analytes^a^.

Target analyte	Age^b^		No. of pregnancies^c^		No. of deliveries^c^		Age at menarche^d^	
	Coef.	*p*-value	Coef.	*p*-value	Coef.	*p*-value	Coef.	*p*-value
Corticosterone	−0.001	0.94	0.003	0.94	−0.007	0.89	0.022	0.66
Hydrocortisone	−0.009	0.73	0.018	0.84	0.024	0.86	−0.025	0.85
Progesterone	−0.027	0.08	0.040	0.45	0.054	0.48	0.023	0.79
Testosterone	−0.002	0.16	−0.007	0.22	−0.012	0.16	0.000	0.97
Pentachlorophenol	0.000	0.93	0.009	0.59	0.005	0.82	0.005	0.84
Caffeine	**0.235**	**3.72E−03**	−0.310	0.28	−0.697	0.09	0.166	0.71
Cotinine	0.060	0.37	−0.068	0.78	0.043	0.90	−0.177	0.72
3,5,6-Trichloro-2-pyridinol	−0.001	0.88	0.011	0.55	−0.006	0.82	**−0.061**	**0.03**
PFHpA	0.001	0.66	−0.001	0.87	0.002	0.81	0.004	0.57
NMeFOSAA	0.002	0.47	0.004	0.72	−0.004	0.80	**0.045**	**0.01**
PFHxS (total)	**0.013**	**9.43E−05**	**−0.029**	**0.01**	**−0.046**	**0.01**	0.027	0.15
PFOA (total)	**0.016**	**3.38E−04**	**−0.034**	**0.04**	−0.040	0.09	0.031	0.22
PFHpS (total)	**0.006**	**3.38E−04**	**−0.013**	**0.05**	−0.016	0.08	**0.029**	**0.01**
PFOS (total)	**0.027**	**0.03**	**−0.090**	**0.05**	−0.104	0.11	**0.170**	**0.02**
NEtFOSAA	0.000	0.95	0.004	0.83	0.000	0.99	**0.059**	**0.04**
FOSAA	−0.008	0.08	−0.002	0.90	−0.016	0.45	**0.048**	**0.04**
FOSA (total)	−0.002	0.32	−0.010	0.21	−0.019	0.10	**0.025**	**0.04**
PFUnDA	0.001	0.53	−0.009	0.07	−0.012	0.11	−0.003	0.71
PFNA	**0.007**	**1.34E−04**	**−0.014**	**0.04**	−0.014	0.16	0.009	0.37
PFDA	**0.002**	**0.05**	−0.007	0.06	−0.009	0.12	0.001	0.85

^a^Transformed values.

^b^Adjusted for: BMI, No. of pregnancies, Sample year, Age at menarche, Tobacco use.

^c^Adjusted for: Age, Birth year, Age at menarche, Tobacco use.

^d^Adjusted for: Age, BMI, Birth year.

Bold values indicate associations that pass a significance threshold of *p*<0.05.

In sensitivity analyses excluding nulliparous women we found the same directional effects as in the main model (data not shown). We did not identify any significant interactions between age and number of pregnancies.

When sum-concentrations for each of the three PFAS co-exposure groups were assessed, Group 1 (PFDA, PFNA and PFUnDA) was positively associated with age (*p* = 0.01), and a negatively associated with number of pregnancies (*p* = 0.04). Group 2 (FOSA, FOSAA and NEtFOSSA) was positively associated with age at menarche (*p* = 0.03), but not with age or parity, and Group 3 (PFOS, PFHpS, PFOA, and PFHxS) displayed positive associations with both age (*p* = 0.001) and age at menarche (*p* = 0.02), and negative associations with both number of pregnancies (*p* = 0.02) and number of deliveries (*p* = 0.04) (Table 3).

**Table 3:** Associations between breast cancer risk factors and groups of PFAS analytes.

PFAS group	Age^a^		No. of pregnancies^b^		No. of deliveries^b^		Age at menarche^c^	
	Coef.	*p*-value	Coef.	*p*-value	Coef.	*p*-value	Coef.	*p*-value
Group 1 (PFDA, PFNA and PFUnDA)	0.01	0.01	−0.03	0.04	−0.03	0.10	0.01	0.72
Group 2 (FOSA, FOSAA and NEtFOSSA)	−0.01	0.37	−0.01	0.84	−0.04	0.52	0.13	0.03
Group 3 (PFOS, PFHpS, PFOA, and PFHxS)	0.06	1.1 × 10^−3^	−0.17	0.02	−0.21	0.04	0.26	0.02

^a^Adjusted for: BMI, No. of pregnancies, Sample year, Age at menarche, Tobacco use.

^b^Adjusted for: Age, Birth year, Age at menarche, Tobacco use.

^c^Adjusted for: Age, BMI, Birth year.

When we assessed longitudinal trends in repeated samples from the same women, several target analytes displayed increasing or declining concentrations over time. This was potentially due to fluctuating levels of these chemicals in the environment as a consequence of compounds being banned during the study period. This was especially evident for the organochlorine pesticide pentachlorophenol, and three PFAS (NEtFOSAA, FOSAA, and total FOSA) all of which decreased between 1987 (when the first samples were collected) and 2006. On the contrary, concentrations of caffeine, TCPy, and of several other PFAS (total PFHxS, total PFOA, PFDA, NMeFOSAA, PFUnDA and PFNA) increased over time (Supplementary Fig. S3).

### Untargeted exposome and associations with breast cancer reproductive risk factors

**Fig. 3:**
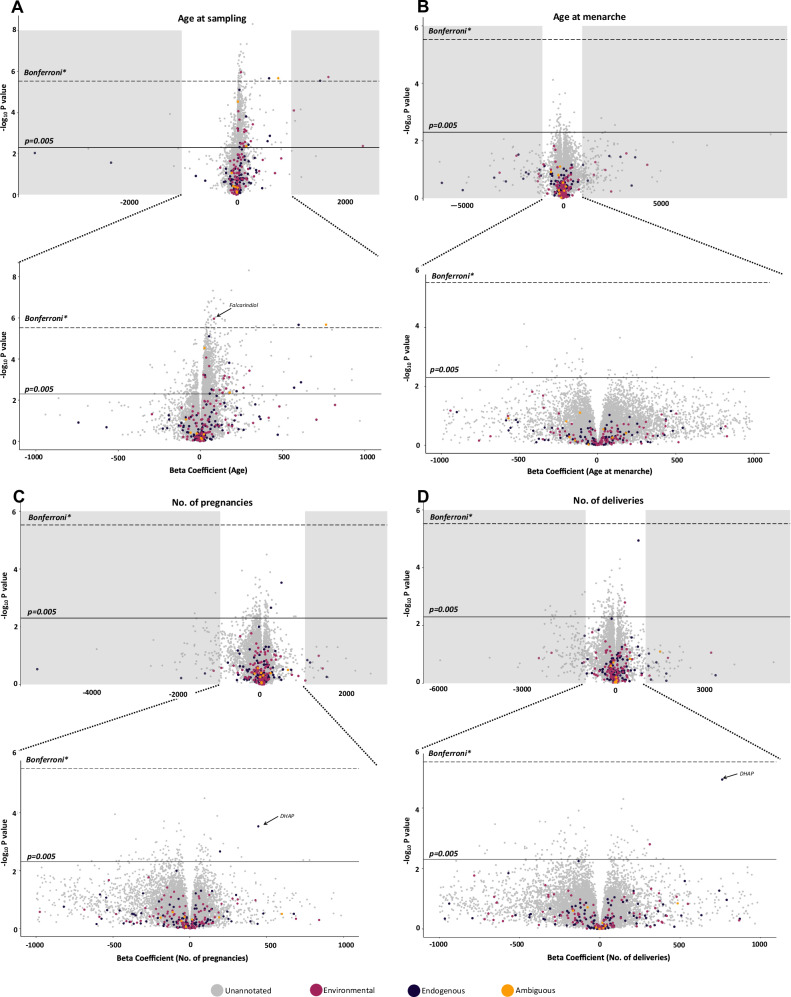
Volcano plots for associations between detected features and different breast cancer risk factors. **A** Associations between age at sampling and features. **B** Associations between age at menarche and features. **C** Associations between no. of pregnancies and features. **D** Associations between no. of deliveries and features. *Bonferroni adjusted threshold = 3.0 × 10^−6^.

Considering the untargeted dataset (excluding the 77 targeted analytes), only a minor fraction could be structurally annotated [25], i.e. 430 out of 94,404 molecular features, including the target analytes (0.06% of all features) and features annotated with high confidence by spectral library match (4 features at Level 1 and 426 features at Level 2). The overall annotation rate was 0.5%, which is typical for untargeted workflows [32]. The annotated features included 246 environmental chemicals, 167 endogenous substances and 17 analytes with ambiguous classification (Supplementary Table S5). The environmental chemicals consisted of 8 subclasses, among which were pesticides, dietary components, food additives, industrial chemicals, and personal care chemicals. Endogenous substances instead consisted of 6 subclasses including fatty acids, bile acids, hormones, and amino acids. Associations with reproductive breast cancer risk factors were evaluated for all features with detection frequency >70%, which included 228 annotated substances (Fig. 3A–D) and 16,530 unannotated features (Fig. 1, and Supplementary Table S5).

In our primary model, evaluating the link between age and chemical burden (adjusted for BMI, number of pregnancies, sample year, age at menarche, and tobacco use) the majority of features increased with increasing age (Fig. 3A). Among the 228 annotated features, 63% (144) increased with age, and 37% (84) decreased with age. After adjusting for multiple testing, 41 features (5 annotated and 36 unannotated) remained significantly positively associated with age (Fig. 3A, Supplementary Table S5).

When assessing associations with parity, no features passed the Bonferroni level adjustments for multiple comparisons. However, in the analyses of both parity measures, one annotated feature i.e. dihydroxyacetone phosphate (DHAP), passed the more stringent *p*-value cutoff of 0.005 (Fig. 3C, D, Supplementary Table S5).

For associations with age at menarche 106 (46%) annotated features displayed negative coefficients and 122 (54%) displayed positive coefficients. No features passed the Bonferroni adjusted threshold and no annotated features passed the cutoff of 0.005 (Fig. 3B and Supplementary Table S5).

## Discussion

In this study, biobanked heparin plasma samples from 100 women (collected between 1987 and 2006) were comprehensively analyzed using a wide scope chemical exposomics protocol [19]. Parallel LC-HRMS targeted and untargeted exposome analysis was successfully demonstrated for the measurement of 55 targeted analytes and 94,349 untargeted features (of which 430 could be annotated at high confidence). In addition, we identified associations between molecular features and reproductive factors known to associate with breast cancer risk, including with age, parity, and age at menarche.

In line with previous studies conducted in the Nordic countries (Sweden, Norway, and Denmark) [33–35], we found that three PFOS precursors (FOSAA, NEtFOSAA, FOSA, all included in our Group 2) decrease in concentration over sampling calendar years. This decrease over time has also been observed in a previous study of Swedish serum samples collected between 1997 and 2012 [36]. However, in the above study they also observed decreasing levels of NMeFOSAA which was not evident in our samples. One potential explanation for this could be that our samples are only collected until year 2007, and altered concentrations related to the phase out of NMeFOSAA might be less evident. Another potential difference related to our discrepant observations of NEtFOSAA and MNeFOSAA levels over time could be the exposure route, as NMeFOSAA and NEtFOSAA are both transformation products from NMeFOSE, and NEtFOSE was previously used in food packaging whereas NMeFOSE was used primarily in textiles, possibly resulting in slower phase out for the latter [37]. Several PFAS chemicals also increased markedly over time, for example PFNA and PFDA, which is consistent with a previous study from 2020 [33], showing that PFDA levels were increasing up until 2004 and PFNA levels up until 2007 before both trends reversed. PFUnDA, which shows a slight increase in our samples, started decreasing after 2008, while PFOS and PFOA were reported to start to decrease in 2001 and 2002 respectively. This is also indicated in our samples where they both seem to be stable over time likely indicative of our samples being collected around the early 2000s.

When assessing associations between targeted analyte concentrations and age, we observed positive associations between PFAS chemicals and age, which was consistent with our original hypothesis. Consistent with the time trends, negative associations could be identified for FOSAA and FOSA, however these did not reach significance after adjusting for sample year. When sample year was substituted with birth year the association was reversed and associations became positive. One possible reason for this could be confounding by birth cohort, which has been suggested to impact associations between age and levels of PFAS chemicals in cross sectional study designs [35, 38]. The fairly large differences in birth years in our dataset may therefore result in difficulty in drawing conclusions on associations between age and chemical burden.

Another compound of note that related to age was caffeine, levels of which seemed to increase significantly with age, which may be explained by decreased metabolic breakdown activity with as a person grows older [39]. Interestingly, the untargeted caffeine metabolites 1-methylxanthine and paraxanthine detected at a confidence Level 2 were also positively associated with age when untargeted analytes were included in the models (*p*-value < 0.005). However, these did not pass Bonferroni corrected thresholds. Among the unannotated features, falcarindiol, a dietary polyacetylenic oxylipin found in carrots [40], was most strongly associated with age and passed the multiple comparison threshold. To our knowledge, no previous studies have linked this compound to increase in age. Despite the low p-value in our analyses, this interesting association will need to be confirmed by other independent studies.

Parity is an established protective factor when it comes to breast cancer risk, although this effect may vary based on menopausal status and breast cancer subtypes [41, 42]. Previous studies have shown that PFAS levels are lower in parous compared to non-parous women [35, 43], most likely due to chemical elimination from the mother to the fetus and through breastfeeding. In our study we had access to information on both number of reported pregnancies (including completed pregnancies as well as pregnancies terminated early) and number of reported deliveries (including completed pregnancies only). We deemed plausible that a number of deliveries would capture chemical excretion both through the fetus and through lactation, whereas number of pregnancies would primarily capture excretion to the fetus, even when there was no breast feeding occurring (due to e.g., early termination). Despite the associations with target analytes being in the same direction for both pregnancies and deliveries, stronger associations were observed when pregnancies were used as the exposure variable. However, this could also be a result of the study design as the women were included based on reported pregnancies (not deliveries).

In line with findings from previous studies, and our original hypothesis, many PFAS analytes associated negatively with parity, and this was especially evident for PFOS, PFHpS, PFOA, and PFHxS when analyzed together as a group (Group 3). Of these, PFOS, PFOA and PFHxS are all classified as substances of very high concern and listed under the Stockholm convention on persistent organic pollutants for global restriction. PFOS is listed since 2009, PFOA since 2019 and PFHxS since 2022 [44, 45].

The feature associating strongest with parity was levels of the endogenous metabolite DHAP, a central metabolic intermediate involved in mitochondrial energy pathways and lipid metabolism. Particularly in mammary glands, DHAP plays a central role in the synthesis of milk fat (triglycerides) [46]. Higher levels of DHAP related to parity might potentially be related to breast feeding although we could not identify any previous studies reporting such associations.

We also examined associations between age at menarche and levels of chemical exposures, with the hypothesis that earlier age at menarche might result in earlier start of using hygiene and cosmetic products therefore resulting in higher levels of some chemicals as these may start to accumulate at an earlier age. There have also been reports on associations between levels of PFOA and PFOS and delayed age at menarche [47], although suggested to be at least partially explained by pharmacokinetics and not due to any direct effects of the compounds [48]. In our data we did identify positive associations between age at menarche and all detected PFOS precursors as well as PFHpS, indicating that a higher age at menarche was associated with higher levels of these compounds. This was contrary to our initial hypothesis but in line with a recent review also identifying positive associations between high exposure to PFHpS (and five other PFAS substances) and higher age at menarche [12]. For this to relate to our study however, we would have to assume that women with the highest exposures as adults also experienced higher exposures during childhood and adolescence.

When analyzing correlations between all the different targeted analytes and the reproductive factors the patterns of positive correlations with age and negative correlations with parity were apparent. Interestingly, progesterone levels were negatively correlated with most PFAS chemicals. One previous study pointed towards PFAS (primarily PFOS and PFOA) being associated with a decreased production of progesterone in women of reproductive ages [49], and our results underscore the importance of this finding and its possible implications for reproductive outcomes.

Study limitations include the relatively small dataset, the fact that nullipara women were, on average, older at sampling than women with previous pregnancies which may distort associations especially related to age. The stringent criteria which included only women donating a blood sample two years after a reported pregnancy might have introduced some selection bias. However, this limitation is outweighed by the advantage of having a similar lead time between pregnancy and sample collection for all women in the study reporting previous pregnancies. Furthermore, measures were performed on heparin plasma although some of the targeted substances are more commonly measured in urine. In addition, we lacked information on age at first pregnancy which is an important reproductive risk factor for breast cancer not investigated within the study. Our samples were collected before any major global bans on these substances took effect and concentrations here may not accurately reflect the current circulation of many of these substances. Finally, it is important to remember that for untargeted features, although the same feature can be compared between samples, intensities cannot be compared between two different features. This can make interpretation difficult which is an important limitation to consider. Finally, since women in the study were primarily from northern European descent, these results may not be generalizable to populations of other ethnic origins or to women living in other geographical areas characterized by different chemical exposures.

A major strength in the study is the exposomics approach which was demonstrated to successfully allow the measure >16,000 untargeted features (including both endogenous and environmental substances) in >70% of samples, and in small volumes of plasma which had been stored for 20 years or more. We also included repeated samples from more than 60 women which made it possible to take time trends into consideration, using a mixed models approach.

Finally, we tested associations between 20 target chemicals and 16,758 untargeted features and different reproductive factors such as parity and age at menarche, identifying related substances that may be of interest for future follow up. Our study hence builds on and strengthens previous knowledge and provides proof that exposomic platforms relying on untargeted methods using HRMS techniques are likely to be of significant use when it comes to identifying disease associated exposures in the future.

## Supplementary information

Supplemental Material

Supplementary Table S1

Supplementary Table S2

Supplementary Table S3

Supplementary Table S4

Supplementary Table S5

## Data Availability

Due to legal restrictions imposed by the Swedish Data Protection Authority, we are not allowed to share individual-level data freely. Instead, any researcher interested in the individual-level data may apply for access at the Biobank Research Unit at Umeå University (https://www.umu.se/en/biobank-research-unit/provsamlingar-och-register/northern-sweden-health-and-disease-study-vip-monica-and-the-mammography-screening-project/). The application will be subject to ethical review and assessment by an expert committee.
