# Supplementary material for: Chemical exposomics in biobanked plasma samples and associations with breast cancer risk factors
Source: J Expo Sci Environ Epidemiol. 2024 Dec 6;35(4):567–77. doi: 10.1038/s41370-024-00736-0 (PMC12234353; doi:10.1038/s41370-024-00736-0)
Supplement: Supplementary file 1 — Supplemental Material [file 41370_2024_736_MOESM1_ESM.docx]

**Supplemental Material**

**Chemical exposomics in biobanked plasma samples and associations with breast cancer risk factors**

Jessica Edlund^1^, Kalliroi Sdougkou^2^, Stefano Papazian^2,3^, Wendy Yi-Ying Wu^1^, Jonathan Martin^2,3^, Sophia Harlid^1*^

*^1^Department of Diagnostics and Intervention, Oncology, Umeå University, Umeå 901 87, Sweden*

^2^*Department of Environmental Science, Science for Life Laboratory, Stockholm University, Stockholm 106 91, Sweden*

*^3^National Facility for Exposomics, Metabolomics Platform, Science for Life Laboratory, Stockholm University, Solna171 65, Sweden*

**Table of Contents: Page**

Figure S1 (Histograms, target analytes, A and B) 2

Figure S2 (Correlation plots) 3

Figure S3 (Line plots, repeated samples) 4

**A**

**B**

**Figure S1.** Histograms of targeted analytes. A) Histograms of original concentrations, B) Histograms after values were transformed by raising to power 1/2 (equivalent to taking the square root).

**A**

**
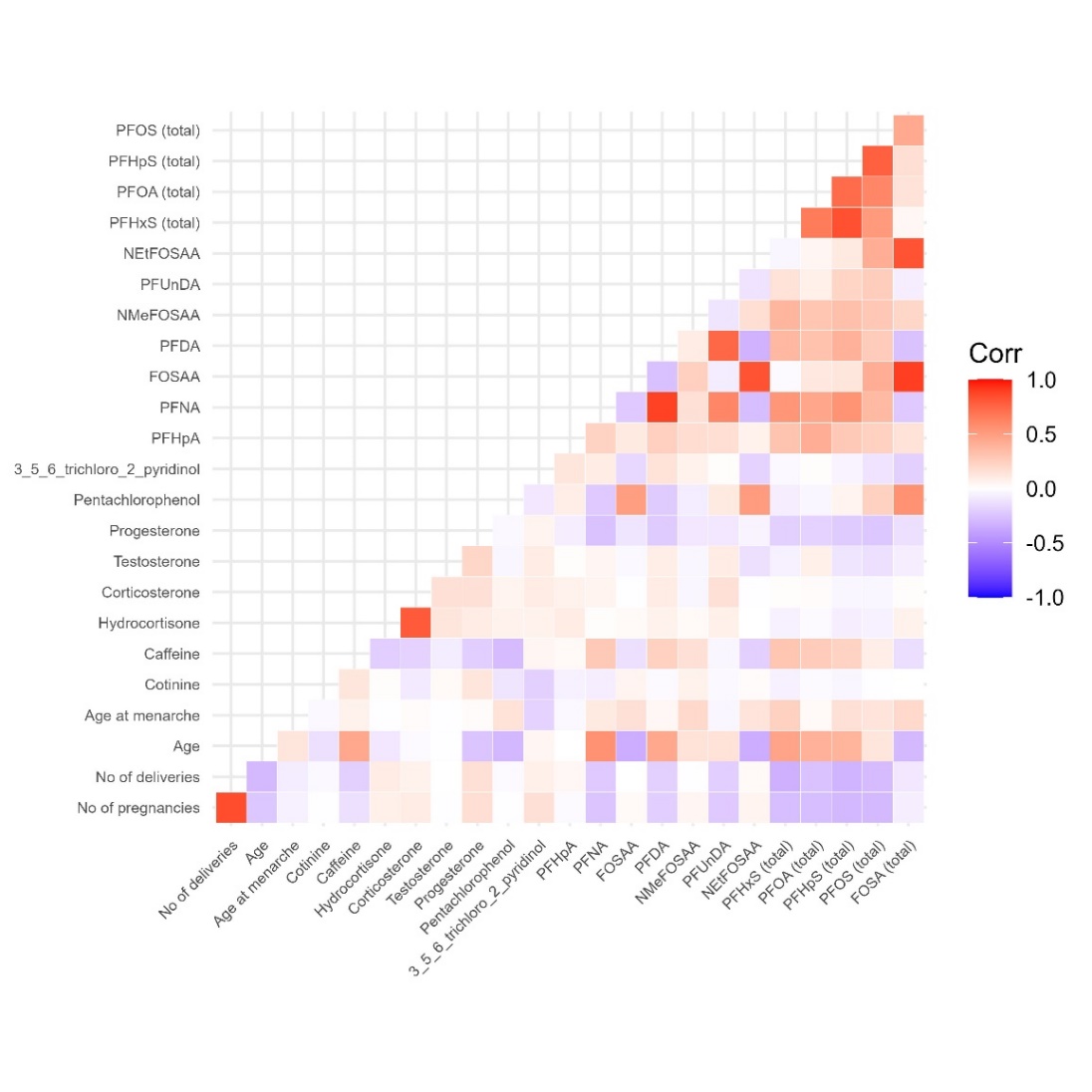
**

**B**

**Figure S2.** Correlations between targeted analytes and reproductive factors. A) Correlations between all chemicals and reproductive factors calculated using Spearman’s correlation test on untransformed values. B) Correlations between different PFAS chemicals calculated using Pearson’s correlation test on transformed values. The three groups correspond to 1) PFDA, PFNA and PFUnDA, 2) FOSA, FOSAA and NEtFOSSA and 3) PFOS, PFHpS, PFOA, and PFHxS.

**Figure S3.** Line plots repeated samples (target analytes), showing trends related to sampling year. Black lines represent the fitted slopes.
