# Supplementary material for: Chemical exposomics in biobanked plasma samples and associations with breast cancer risk factors
Source: J Expo Sci Environ Epidemiol. 2024 Dec 6;35(4):567–77. doi: 10.1038/s41370-024-00736-0 (PMC12234353; doi:10.1038/s41370-024-00736-0)
Supplement: Supplementary file 2 — Supplementary Table S1 [file 41370_2024_736_MOESM2_ESM.pdf]

Supplementary Table S1. Information on labelled standard compounds used in this study.

Compound name	CAS-number	Supplier	Purity	Use
Acephate-acetyl-d3	2140327-70-2	Supelco	100%	internal standard, spiked before extraction
2,4-Dichlorophenoxyacetic acid-13C6	150907-52-1	Toronto Research Chemicals	99%	internal standard, spiked before extraction
4-Methylnitrosamino-1-3-pyridyl-1-butanol-d5 (NNAL-d5)	1794885-45-2	Toronto Research Chemicals	100%	internal standard, spiked before extraction
4-Methylhippuric acid-d7	1216588-60-1	Toronto Research Chemicals	98%	internal standard, spiked before extraction
Atrazine-d5	163165-75-1	Merck	100%	internal standard, spiked before extraction
4-Nitrophenol-2,3,5,6-d4 (Nitrophenol-d4)	93951-79-2	Merck	>98%	internal standard, spiked before extraction
Bisphenol A-13C12	263261-65-0	Toronto Research Chemicals	98%	internal standard, spiked before extraction
Daidzein-d6	291759-05-2	Toronto Research Chemicals	98%	internal standard, spiked before extraction
N,N-Diethyl-3-methylbenzamide-d10 (DEET-d10)	1215576-01-4	Toronto Research Chemicals	98%	internal standard, spiked before extraction
Diclofenac -d4	153466-65-0	Santa Cruz Biotechnology	98%	internal standard, spiked before extraction
Diphenyl phosphate-d10	1477494-97-5	Toronto Research Chemicals	95%	internal standard, spiked before extraction
Monoethyl phthalate-d4	1219806-03-7	Toronto Research Chemicals	98%	internal standard, spiked before extraction
Monoisobutyl phthalate -d4	1219802-26-2	Toronto Research Chemicals	100%	internal standard, spiked before extraction
Norharman-d7	1216503-21-7	Toronto Research Chemicals	98%	internal standard, spiked before extraction
Oxybenzone-d3	not provided	Toronto Research Chemicals	98%	internal standard, spiked before extraction
Pentachlorophenol-13C6	85380-74-1	Toronto Research Chemicals	98%	internal standard, spiked before extraction
Propylparaben-d7	1246820-92-7	Toronto Research Chemicals	98%	internal standard, spiked before extraction
Cotinine-13C-d3	1215842-75-3	Toronto Research Chemicals	98%	internal standard, spiked before extraction
Enterolactone-13C3	918502-72-4	Toronto Research Chemicals	95%	internal standard, spiked before extraction
Triclosan-13C6	not provided	Toronto Research Chemicals	99%	internal standard, spiked before extraction
Perfluoro-n-[13C4]butanoic acid (MPFBA)*	not provided	Wellington Laboratories	>98%	internal standard, spiked before extraction
Perfluoro-n-[13C5]pentanoic acid (M5PFPeA)*	not provided	Wellington Laboratories	>98%	internal standard, spiked before extraction
Perfluoro-n-[1,2,3,4,6-13C5]hexanoic acid (M5PFHxA)*	not provided	Wellington Laboratories	>98%	internal standard, spiked before extraction
Perfluoro-n-[1,2,3,4-13C4]heptanoic acid (M4PFHpA)*	not provided	Wellington Laboratories	>98%	internal standard, spiked before extraction
Perfluoro-n-[13C8]octanoic acid (M8PFOA)*	not provided	Wellington Laboratories	>98%	internal standard, spiked before extraction
Perfluoro-n-[13C9]nonanoic acid (M9PFNA)*	not provided	Wellington Laboratories	>98%	internal standard, spiked before extraction
Perfluoro-n-[1,2,3,4,5,6-13C6]decanoic acid (M6PFDA)*	not provided	Wellington Laboratories	>98%	internal standard, spiked before extraction
Perfluoro-n-[1,2,3,4,5,6,7-13C7]undecanoic acid (M7PFUdA)*	not provided	Wellington Laboratories	>98%	internal standard, spiked before extraction
Perfluoro-n-[1,2-13C2]dodecanoic acid (MPFDoA)*	not provided	Wellington Laboratories	>98%	internal standard, spiked before extraction
Perfluoro-n-[1,2-13C2]tetradecanoic acid (M2PFTeDA)*	not provided	Wellington Laboratories	>98%	internal standard, spiked before extraction
Sodium perfluoro-1-[2,3,4-13C3]butanesulfonate (M3PFBS)*	not provided	Wellington Laboratories	>98%	internal standard, spiked before extraction
Sodium perfluoro-1-[1,2,3-13C3]hexanesulfonate (M3PFHxS)*	not provided	Wellington Laboratories	>98%	internal standard, spiked before extraction
Sodium perfluoro-1-[13C8]octanesulfonate (M8PFOS)*	not provided	Wellington Laboratories	>98%	internal standard, spiked before extraction
Perfluoro-1-[13C8]octanesulfonamide (M8FOSA)	not provided	Wellington Laboratories	>98%	internal standard, spiked before extraction
Diuron-d6	1007536-67-5	Santa Cruz Biotechnology	99%	volumetric standard, spiked after extraction

* MIX: MPFACCES
