# Supplementary material for: Chemical exposomics in biobanked plasma samples and associations with breast cancer risk factors
Source: J Expo Sci Environ Epidemiol. 2024 Dec 6;35(4):567–77. doi: 10.1038/s41370-024-00736-0 (PMC12234353; doi:10.1038/s41370-024-00736-0)
Supplement: Supplementary file 3 — Supplementary Table S2 [file 41370_2024_736_MOESM3_ESM.pdf]

Supplementary Table S2. Target analytes and results of method validation.

Compound Class	Compound name	RT (min)	Log P*	ESI mode	MLOQ (ng/ml)
Flame Retardants and Metabolites	Bis(1,3-dichloro-2-propyl) phosphate	11.6	2.2	negative	0.50
Flame Retardants and Metabolites	Diphenyl phosphate	10.4	2.9	negative	0.50
Flame Retardants and Metabolites	Tetrabromobisphenol A (TBBPA)	16.2	7.2	negative	5.00
Flame Retardants and Metabolites	Tris(2-butoxyethyl) phosphate	16.4	3.0	positive	0.01
Food Related Compounds	Acesulfame	2.5	-1.3	negative	0.10
Food Related Compounds	Caffeine	7.1	-0.1	positive	0.03
Fungicides and Metabolites	Pentachlorophenol	14.6	5.1	negative	0.05
Herbicides and Metabolites	2,4-Dichlorophenoxyacetic acid	10.3	2.8	negative	0.50
Herbicides and Metabolites	4-Methylhippuric acid	7.0	1.0	negative	1.00
Herbicides and Metabolites	Atrazine	12.7	2.6	positive	0.03
Herbicides and Metabolites	Atrazine desethyl	9.5	1.5	positive	0.50
Herbicides and Metabolites	Atrazine desethyl-2-hydroxy	5.1	-2.3	positive	0.50
Herbicides and Metabolites	Atrazine desisopropyl	7.7	1.2	positive	0.50
Herbicides and Metabolites	Atrazine-2-hydroxy	10.1	-1.3	positive	0.02
Herbicides and Metabolites	Diuron	12.9	2.7	negative	0.01
Heterocyclic Amines	Norharman	11.0	2.1	negative	0.40
Insect Repellent and Metabolites	Diazinon	15.4	3.8	positive	0.01
Insect Repellent and Metabolites	N,N-Diethyl-3-methylbenzamide (DEET)	12.8	2.2	positive	0.01
Neonicotinoids	Imidacloprid	8.1	0.6	positive	0.01
Neonicotinoids	Thiamethoxam	7.0	0.8	positive	0.11
Organophosphorus Insecticides and Specific Metabolites	3,5,6-Trichloro-2-pyridinol (TCPy)	11.6	1.2	negative	0.05
Organophosphorus Insecticides and Specific Metabolites	4-Nitrophenol	9.2	1.9	negative	0.02
Organophosphorus Insecticides and Specific Metabolites	Acephate	4.5	-0.9	positive	1.00
Organophosphorus Insecticides and Specific Metabolites	Dimethoate	8.8	0.8	positive	0.01
Organophosphorus Insecticides and Specific Metabolites	Malathion	14.1	2.4	positive	0.01
Organophosphorus Insecticides and Specific Metabolites	Methamidophos	3.2	-0.8	positive	0.50
Perfluoroalkyl and Polyfluoroalkyl Substances (PFAS)	Perfluoro-1-butanesulfonate (PFBS)	10.9	-3.6	negative	0.02
Perfluoroalkyl and Polyfluoroalkyl Substances (PFAS)	Perfluoro-1-decanesulfonate (PFDS)	15.7	-0.5	negative	0.03
Perfluoroalkyl and Polyfluoroalkyl Substances (PFAS)	Perfluoro-1-heptanesulfonate (PFHpS)	14.1	-2.0	negative	0.01
Perfluoroalkyl and Polyfluoroalkyl Substances (PFAS)	Perfluoro-1-hexanesulfonate (PFHxS)	13.3	-2.5	negative	0.03
Perfluoroalkyl and Polyfluoroalkyl Substances (PFAS)	Perfluoro-1-nonanesulfonate (PFNS)	15.2	-1.0	negative	0.04
Perfluoroalkyl and Polyfluoroalkyl Substances (PFAS)	Perfluoro-1-octanesulfonate (PFOS)	14.7	-1.5	negative	0.03
Perfluoroalkyl and Polyfluoroalkyl Substances (PFAS)	Perfluoro-1-pentanesulfonate (PFPeS)	12.3	-3.0	negative	0.03
Perfluoroalkyl and Polyfluoroalkyl Substances (PFAS)	Perfluoro-n-butanoic acid (PFBA)	7.3	-2.8	negative	0.30
Perfluoroalkyl and Polyfluoroalkyl Substances (PFAS)	Perfluoro-n-decanoic acid (PFDA)	15.2	0.3	negative	0.05
Perfluoroalkyl and Polyfluoroalkyl Substances (PFAS)	Perfluoro-n-dodecanoic acid (PFDoDA)	16.1	not available	negative	0.08
Perfluoroalkyl and Polyfluoroalkyl Substances (PFAS)	Perfluoro-n-heptanoic acid (PFHpA)	13.2	-2.3	negative	0.03
Perfluoroalkyl and Polyfluoroalkyl Substances (PFAS)	Perfluoro-n-hexanoic acid (PFHxA)	12.2	-1.8	negative	0.03
Perfluoroalkyl and Polyfluoroalkyl Substances (PFAS)	Perfluoro-n-nonanoic acid (PFNA)	14.7	-0.3	negative	0.03
Perfluoroalkyl and Polyfluoroalkyl Substances (PFAS)	Perfluoro-n-octanoic acid (PFOA)	14.1	-0.8	negative	0.03
Perfluoroalkyl and Polyfluoroalkyl Substances (PFAS)	Perfluoro-n-pentanoic acid (PFPeA)	10.5	-2.3	negative	0.03
Perfluoroalkyl and Polyfluoroalkyl Substances (PFAS)	Perfluoro-n-tetradecanoic acid (PFTeDA)	16.6	not available	negative	0.07
Perfluoroalkyl and Polyfluoroalkyl Substances (PFAS)	Perfluoro-n-tridecanoic acid (PFTrDA)	16.4	not available	negative	0.04
Perfluoroalkyl and Polyfluoroalkyl Substances (PFAS)	Perfluoro-n-undecanoic acid (PFUnDA)	15.7	0.8	negative	0.05
Perfluoroalkyl and Polyfluoroalkyl Substances (PFAS)	Perfluorooctane sulfonamide (FOSA)	16.0	5.8	negative	0.05

Perfluoroalkyl and Polyfluoroalkyl Substances (PFAS)	Perfluoro-1-octanesulfonamidoacetic acid (FOSAA)	15.2	5.5	negative	0.10
Perfluoroalkyl and Polyfluoroalkyl Substances (PFAS)	N-methylperfluoro-1-butanesulfonamide (NMeFBSA)	14.3	3.6	negative	0.05
Perfluoroalkyl and Polyfluoroalkyl Substances (PFAS)	N-ethylperfluoro-1-octanesulfonamicoacetic acid (NEtFOSA	15.4	6.2	negative	0.05
Perfluoroalkyl and Polyfluoroalkyl Substances (PFAS)	N-methylperfluoro-1-octanesulfonamidoacetic acid (NMeF	15.6	5.7	negative	0.05
Personal Care and Consumer Product Chemicals and Metabolites	2,5-Dichlorophenol	10.3	3.1	negative	0.40
Personal Care and Consumer Product Chemicals and Metabolites	Bisphenol A (BPA)	13.0	3.3	negative	0.40
Personal Care and Consumer Product Chemicals and Metabolites	Methylparaben	9.8	2.0	negative	0.05
Personal Care and Consumer Product Chemicals and Metabolites	Octocrylene	17.4	6.9	positive	5.00
Personal Care and Consumer Product Chemicals and Metabolites	Oxybenzone	15.0	3.8	positive	0.01
Personal Care and Consumer Product Chemicals and Metabolites	Phenylparaben	13.3	3.2	negative	0.50
Personal Care and Consumer Product Chemicals and Metabolites	Propylparaben	12.8	3.0	negative	0.03
Personal Care and Consumer Product Chemicals and Metabolites	Triclocarban	16.1	4.9	negative	0.10
Personal Care and Consumer Product Chemicals and Metabolites	Triclosan	16.3	4.8	negative	0.08
Pesticides	Carbofuran	11.5	2.3	positive	0.05
Pesticides	Clorpyrifos	16.9	5.0	positive	0.03
Pharmaceuticals	Diclofenac	13.5	0.7	negative	0.50
Pharmaceuticals	Ibuprofen	14.1	3.8	negative	0.50
Pharmaceuticals / Aniline metabolites	Paracetamol	4.9	0.5	positive	0.35
Phthalate and Phthalate Alternative Metabolites	Mono(2-ethyl-5-hydroxyhexyl) phthalate	10.9	3.2	negative	2.50
Phthalate and Phthalate Alternative Metabolites	Mono(5-carboxy-2-ethylpentyl) phthalate	10.2	3.5	negative	0.50
Phthalate and Phthalate Alternative Metabolites	Monoethyl phthalate	6.7	2.8	negative	0.50
Phthalate and Phthalate Alternative Metabolites	Monoisobutyl phthalate	9.8	2.8	negative	1.00
Phytoestrogens and Metabolites	Daidzein	11.0	2.6	negative	0.05
Phytoestrogens and Metabolites	Enterolactone	11.6	2.7	negative	0.05
Phytoestrogens and Metabolites	Equol	11.5	3.7	negative	0.40
Polycyclic Aromatic Hydrocarbon Metabolites	1-Naphthol	12.5	2.9	negative	0.40
Polycyclic Aromatic Hydrocarbon Metabolites	2-Naphthol	12.2	2.7	negative	0.1
Pyrethroids and Metabolites	Bioallethrin	16.6	4.8	positive	0.33
Pyrethroids and Metabolites	3-Phenoxybenzoic acid	11.7	3.9	negative	0.50
Steroid Hormones	Corticosterone	13.1	2.0	positive	0.01
Steroid Hormones	Estradiol	13.7	3.9	negative	0.50
Steroid hormones	Hydocortisone	12.2	1.6	positive	0.01
Steroid Hormones	Progesterone	15.3	3.7	positive	0.01
Steroid Hormones	Testosterone	14.1	3.3	positive	0.01
Tobacco Alkaloids, Nitrosamines and Metabolites	4-(Methylnitrosamino)-1-(3-pyridyl)-1-butanol (NNAL)	6.9	-0.2	positive	0.50
Tobacco Alkaloids, Nitrosamines and Metabolites	Cotinine	6.6	0.1	positive	0.05

MLOQ: Method Limit of Quantification
RT: Retention Time

*Log P values are estimated from EPA EPI Suite software, besides the estimations for the anionic perfluoroalkyl acids included in the class of perfluoroalkyl and polyfluoroalkyl substances, which are from Hidalgo and Mora-Diez (Hidalgo, A.; Mora-Diez, N. Novel approach for predicting partition coefficients of linear perfluorinated compounds. Theor. Chem. Acc. 2016, 135 (1), 18.).
