# Supplementary material for: Chemical exposomics in biobanked plasma samples and associations with breast cancer risk factors
Source: J Expo Sci Environ Epidemiol. 2024 Dec 6;35(4):567–77. doi: 10.1038/s41370-024-00736-0 (PMC12234353; doi:10.1038/s41370-024-00736-0)
Supplement: Supplementary file 4 — Supplementary Table S3 [file 41370_2024_736_MOESM4_ESM.pdf]

Supplementary Table S3. MSDIAL parameters for chromatographic alignment, spectral deconvolution and peak integration.

Step	Settings
Samples included in the processing	S3WP individual samples, pooled Swedish plasma samples (spiked and unspiked), procedural blanks
Data Collection	MS1 mass range begin: 50<br>MS1 mass range end: 1000<br>MS/MS mass range begin: 50<br>MS/MS mass range end: 1000<br>Processing threads: 36
Mass accuracy	MS1 tolerance: 0.0012 Da<br>MS2 tolerance: 0.005 Da
Maximum charged number	2
Consider Cl and Br elements	TRUE
Minimum peak width	9
Minimum peak height	85,000
Mass slice width	0.05
Smoothing method	Linear weighted moving average
Smoothing level	3
Minimum peak width	8
Exclusion mass list	No
Sigma window value	0.5
MS2Dec amplitude cut off	0
Exclude after precursor ion	TRUE
Keep isotope until	3
Keep original precursor isotopes	TRUE
MSP file (library)	MassBank of North America (MoNA), GNPS, MassBankEU
Accurate mass tolerance (MS1):	0.005 Da
Accurate mass tolerance (MS2):	0.01 Da
Identification score cut off:	70
Use retention time for scoring/filtering:	FALSE
Adduct ion setting	ESI+: [M+H]+<br>[M+NH4]+<br>[M+Na]+<br>[M+H-H2O]+<br>[M+H-2H2O]+<br>[2M+H]+<br>[2M+NH4]+<br>ESI-: [M-H]-, [M-H2O-H]-
Reference File	pooled Swedish plasma spiked with all native target analytes
Retention time tolerance	0.25 min
MS1 tolerance	0.0012 Da
Retention time factor	0.5
MS1 factor	0.5
Peak count filter	0%
N% detected in at least one group	0%
Remove feature based on peak height fold-change	TRUE
Sample max / blank average	5-fold change
Keep identified and annotated metabolites	FALSE
Keep “suggested (w/o MS2)” metabolite features	FALSE
Keep removable features and assign the tag for checking	TRUE
Gap filling by compulsion	TRUE
Exported table for further data analysis and visualisations	Alignment result, Raw data matrix (area)
