# Supplementary material for: Chemical exposomics in biobanked plasma samples and associations with breast cancer risk factors
Source: J Expo Sci Environ Epidemiol. 2024 Dec 6;35(4):567–77. doi: 10.1038/s41370-024-00736-0 (PMC12234353; doi:10.1038/s41370-024-00736-0)
Supplement: Supplementary file 5 — Supplementary Table S4 [file 41370_2024_736_MOESM5_ESM.pdf]

Supplementary Table S4. Target analytes detected and quantified in authentic human plasma samples.

Compound Class	Analyte	Nondetect (%)	Nondetect and detect < MLOQ (%)	Lowest quantified concentration (ng/ml)	Highest quantified concentration (ng/ml)	Included in statistical analysis (yes/no)
Flame Retardants and Metabolites	Bis(1,3-dichloro-2-propyl) phosphate	81%	94%	0.13	7.3	no
Flame Retardants and Metabolites	Diphenyl phosphate	92%	92%	0.13	0.70	no
Flame Retardants and Metabolites	Tris(2-butoxyethyl) phosphate	96%	96%	0.06	0.69	no
Food Related Compounds	Caffeine	1%	1%	0.01	2651	yes
Food Related Compounds	Acesulfame	76%	86%	0.13	66	no
Fungicides and Metabolites	Pentachlorophenol	0%	0%	0.14	8.0	yes
Herbicides and Metabolites	Atrazine	79%	96%	0.01	0.23	no
Herbicides and Metabolites	4-Methylhippuric acid	81%	87%	0.25	7.4	no
Herbicides and Metabolites	2,4-Dichlorophenoxyacetic acid	89%	97%	0.13	0.54	no
Neonicotinoids	Imidacloprid	97%	99%	0.003	0.15	no
Organophosphorus Insecticides and Specific Metabolites	3,5,6-Trichloro-2-pyridinol (TCPy)	6%	8%	0.01	24	yes
Perfluoroalkyl and Polyfluoroalkyl Substances (PFAS)	Perfluoro-n-decanoic acid (PFDA)	0%	0%	0.05	0.81	yes
Perfluoroalkyl and Polyfluoroalkyl Substances (PFAS)	Perfluoro-n-nonanoic acid (PFNA)	0%	0%	0.15	1.7	yes
Perfluoroalkyl and Polyfluoroalkyl Substances (PFAS)	Perfluoro-1-heptanesulfonate (PFHpS)	0%	0%	0.06	2.2	yes*
Perfluoroalkyl and Polyfluoroalkyl Substances (PFAS)	total branched PFOS (br-PFHpS)	3%	11%	0.003	0.3	
Perfluoroalkyl and Polyfluoroalkyl Substances (PFAS)	N-methylperfluoro-1-octanesulfonamidoacetic acid (NMeFOSAA)	0%	1%	0.03	2.9	yes
Perfluoroalkyl and Polyfluoroalkyl Substances (PFAS)	Perfluoro-1-hexanesulfonate (PFHxS)	0%	0%	0.24	4.7	yes*
Perfluoroalkyl and Polyfluoroalkyl Substances (PFAS)	total branched PFHxS (br-PFHxS)	4%	41%	0.01	0.1	
Perfluoroalkyl and Polyfluoroalkyl Substances (PFAS)	Perfluoro-n-octanoic acid (PFOA)	0%	0%	0.4	7.6	yes*
Perfluoroalkyl and Polyfluoroalkyl Substances (PFAS)	total branched PFOA (br-PFOA)	6%	14%	0.0	0.6	
Perfluoroalkyl and Polyfluoroalkyl Substances (PFAS)	Perfluoro-1-octanesulfonate (PFOS)	0%	0%	2.9	64	yes*
Perfluoroalkyl and Polyfluoroalkyl Substances (PFAS)	total branched PFOS (br-PFOS)	0%	0%	1.0	40.1	
Perfluoroalkyl and Polyfluoroalkyl Substances (PFAS)	Perfluoro-n-undecanoic acid (PFUnDA)	1%	1%	0.01	0.88	yes
Perfluoroalkyl and Polyfluoroalkyl Substances (PFAS)	Perfluoro-1-octanesulfonamidoacetic acid (FOSAA)	4%	7%	0.01	7.8	yes
Perfluoroalkyl and Polyfluoroalkyl Substances (PFAS)	Perfluorooctane sulfonamide (FOSA)	4%	12%	0.01	1.6	yes*
Perfluoroalkyl and Polyfluoroalkyl Substances (PFAS)	total branched FOSA (br-FOSA)	12%	31%	0.01	0.9	
Perfluoroalkyl and Polyfluoroalkyl Substances (PFAS)	N-ethylperfluoro-1-octanesulfonamicoacetic acid (NEtFOSAA)	10%	10%	0.01	12	yes
Perfluoroalkyl and Polyfluoroalkyl Substances (PFAS)	Perfluoro-n-heptanoic acid (PFHpA)	14%	30%	0.01	0.82	yes
Perfluoroalkyl and Polyfluoroalkyl Substances (PFAS)	Perfluoro-n-pentanoic acid (PFPeA)	48%	63%	0.01	0.10	no
Perfluoroalkyl and Polyfluoroalkyl Substances (PFAS)	Perfluoro-1-pentanesulfonate (PFPeS)	48%	79%	0.01	0.14	no
Perfluoroalkyl and Polyfluoroalkyl Substances (PFAS)	Perfluoro-1-butanesulfonate (PFBS)	78%	94%	0.005	0.07	no
Perfluoroalkyl and Polyfluoroalkyl Substances (PFAS)	N-methylperfluoro-1-butanesulfonamide (NMeFBSA)	80%	86%	0.003	0.02	no
Perfluoroalkyl and Polyfluoroalkyl Substances (PFAS)	Perfluoro-1-decanesulfonate (PFDS)	80%	91%	0.01	3.8	no
Perfluoroalkyl and Polyfluoroalkyl Substances (PFAS)	Perfluoro-n-dodecanoic acid (PFDoDA)	82%	98%	0.02	0.12	no
Perfluoroalkyl and Polyfluoroalkyl Substances (PFAS)	Perfluoro-1-nonanesulfonate (PFNS)	98%	99%	0.01	0.10	no
Personal Care and Consumer Product Chemicals and Metabolites	Methylparaben	57%	57%	0.01	28	no
Personal Care and Consumer Product Chemicals and Metabolites	Triclosan	60%	79%	0.02	0.71	no
Personal Care and Consumer Product Chemicals and Metabolites	Propylparaben	75%	75%	0.01	11	no
Personal Care and Consumer Product Chemicals and Metabolites	Oxybenzone	91%	91%	0.10	2.9	no
Personal Care and Consumer Product Chemicals and Metabolites	2,5-Dichlorophenol	94%	96%	0.10	0.61	no
Pharmaceuticals	Diclofenac	98%	99%	0.13	151	no
Pharmaceuticals	Ibuprofen	57%	60%	0.13	44858	no
Pharmaceuticals / Aniline metabolites	Paracetamol	68%	73%	0.09	4112	no
Phthalates and Plasticizers Metabolites	Monobutyl phthalate	49%	49%	0.25	88	no
Phthalates and Plasticizers Metabolites	Mono(5-carboxy-2-ethylpentyl) phthalate	64%	78%	0.13	5.4	no
Phthalates and Plasticizers Metabolites	Monoethyl phthalate	75%	75%	0.13	78	no
Phthalates and Plasticizers Metabolites	Monoisobutyl phthalate	99%	99%	0.25	7.2	no
Phytoestrogens and Metabolites	Enterolactone	73%	83%	0.01	0.36	no
Phytoestrogens and Metabolites	Daidzein	76%	89%	0.01	0.21	no
Phytoestrogens and Metabolites	Equol	92%	98%	0.03	0.48	no
Steroid Hormones	Corticosterone	0%	0%	0.85	33	yes
Steroid hormones	Hydrocortisone	0%	0%	45	351	yes
Steroid Hormones	Testosterone	7%	7%	0.001	0.46	yes
Steroid Hormones	Progesterone	31%	31%	0.002	23	yes
Tobacco Alkaloids, Nitrosamines and Metabolites	Cotinine	36%	42%	0.01	773	yes

* included in statistical analysis as the sum of linear and branched isomers
