# Supplementary material for: Chemical exposomics in biobanked plasma samples and associations with breast cancer risk factors
Source: J Expo Sci Environ Epidemiol. 2024 Dec 6;35(4):567–77. doi: 10.1038/s41370-024-00736-0 (PMC12234353; doi:10.1038/s41370-024-00736-0)
Supplement: Supplementary file 6 — Supplementary Table S5 [file 41370_2024_736_MOESM6_ESM.pdf]

Supplementary Table S5. Level 1 and Level 2 annotated features in authentic human plasma samples.

Annotation	Ionization	Rt (min)	m/z	Confidence level	Class	Subclass	Included in statistical analysis (yes/no)	Coefficient (Age)	p-value (Age)	Coefficient (No of pregnancies)	p-value (No of pregnancies)	Coefficient (No of deliveries)	p-value (No of deliveries)	Coefficient (Age at menarche)	p-value (Age at menarche)
DHAP	neg	7.37	168.99	Level 2	Endogenous	other endogenous metabolites	Yes	-57.87	0.06	446.29	0.00	763.13	0.00	-16.40	0.93
Estrone-3-sulfate	neg	11.30	349.11	Level 2	Endogenous	hormones and metabolites	Yes	-15.37	0.37	197.01	0.00	144.01	0.13	-13.84	0.89
(1'S,2'S)-3',11'-dihydroxy-1',2',5'-trimethyl-8'-oxaspiro[oxirane-2,12'-tricyclo[7.2.1.0,?]dodecan]-5'-en-4'-one	neg	10.47	279.12	Level 2	Endogenous	other endogenous metabolites	Yes	7.87	0.39	-86.52	0.01	-133.11	0.01	-49.93	0.34
Nonaethylene glycol	pos	8.28	415.25	Level 2	Environmental	industrial chemicals and metabolites	Yes	26.53	0.40	-267.99	0.02	-340.07	0.04	64.63	0.71
Hexaethylene glycol	pos	6.79	283.18	Level 2	Environmental	industrial chemicals and metabolites	Yes	-73.18	0.25	-527.75	0.02	-783.00	0.02	434.01	0.23
Decaethylene glycol	pos	8.65	459.28	Level 2	Environmental	industrial chemicals and metabolites	Yes	28.38	0.15	-147.35	0.04	-196.62	0.05	46.68	0.67
17alpha-Hydroxyprogesterone	neg	14.31	329.21	Level 2	Endogenous	hormones and metabolites	Yes	-11.79	0.26	72.98	0.05	90.90	0.09	-22.43	0.70
3?-Hydroxy-7 Ketolithocholic Acid	pos	14.00	391.28	Level 2	Endogenous	bile acids	Yes	-1.33	0.95	145.11	0.05	168.27	0.11	-104.70	0.36
Undecaethylene glycol	pos	8.98	503.31	Level 2	Environmental	industrial chemicals and metabolites	Yes	29.13	0.05	-102.35	0.05	-123.40	0.11	12.06	0.88
2-[(4-methoxyphenyl)carbonylamino]acetic acid	pos	5.95	210.08	Level 2	Endogenous	other endogenous metabolites	Yes	5.00	0.91	-304.39	0.06	-569.37	0.01	469.25	0.07
5-methoxytryptamine	neg	10.11	189.10	Level 2	Endogenous	aminoacids and derivatives	Yes	0.65	0.90	38.80	0.06	48.29	0.10	-33.43	0.29
indole-3-carboxaldehyde	pos	9.14	146.06	Level 2	Endogenous	other endogenous metabolites	Yes	111.89	0.19	-584.81	0.07	-1037.45	0.02	544.06	0.26
Biliverdin	neg	14.08	581.24	Level 2	Environmental	foods and additives	Yes	-44.00	0.02	126.90	0.07	311.27	0.00	27.37	0.80
Lithocholic Acid	neg	16.48	375.29	Level 2	Endogenous	bile acids	Yes	8.41	0.85	301.72	0.07	529.65	0.03	-35.01	0.89
Linalool	pos	14.02	155.14	Level 2	Environmental	personal care chemicals and metabolites	Yes	-48.26	0.06	-166.36	0.07	-135.99	0.31	-248.45	0.08
Ioxynil	neg	10.50	369.82	Level 2	Environmental	pesticides and metabolites	Yes	-13.20	0.04	-43.03	0.07	-36.93	0.29	-6.19	0.87
1-[benzyloxycarbonyl]pyrrolidine-2-carboxylic acid	pos	11.22	250.11	Level 2	Environmental	industrial chemicals and metabolites	Yes	35.34	0.00	58.66	0.08	75.00	0.12	16.99	0.74
4-Hydroxycoumarin_3	pos	10.18	163.04	Level 2	Environmental	foods and additives	Yes	-15.79	0.44	-122.40	0.08	-178.78	0.08	-24.69	0.82
Indolelactic acid	pos	6.00	206.08	Level 2	Endogenous	aminoacids and derivatives	Yes	-73.45	0.37	-544.64	0.09	-438.29	0.34	480.53	0.32
Pygenic acid B	neg	15.46	487.34	Level 2	Environmental	plant metabolites and natural products	Yes	-5.38	0.61	62.16	0.09	36.93	0.49	-13.32	0.82
Coniferaldehyde	pos	9.90	179.07	Level 2	Environmental	foods and additives	Yes	141.82	0.00	278.21	0.09	372.98	0.12	-254.73	0.28
Indole	pos	5.98	118.07	Level 2	Endogenous	other endogenous metabolites	Yes	-22.47	0.05	-73.02	0.09	-38.66	0.54	14.03	0.83
18b-glycyrrhetinic acid	neg	16.84	469.33	Level 2	Environmental	foods and additives	Yes	9.17	0.86	312.35	0.10	345.44	0.21	25.51	0.93
Chlorothalonil-4-hydroxy	neg	11.44	244.91	Level 2	Environmental	pesticides and metabolites	Yes	14.09	0.64	-183.28	0.10	-287.00	0.07	-1.64	0.99
PhenylPropionicAcid	neg	9.37	149.06	Level 2	Environmental	foods and additives	Yes	321.47	0.13	1321.14	0.10	1747.80	0.13	827.52	0.50
N-(cinnamoyl)glycine	pos	7.76	206.08	Level 2	Environmental	foods and additives	Yes	93.47	0.17	427.80	0.11	549.20	0.15	318.25	0.42
Glycerol 1-myristate	pos	17.64	303.25	Level 2	Endogenous	fatty acids and derivatives	Yes	99.47	0.01	-200.45	0.12	-194.74	0.29	-224.30	0.26
Hexanedioic acid	neg	3.39	145.05	Level 2	Environmental	other contaminants and metabolites	Yes	25.17	0.64	310.50	0.14	248.00	0.41	-241.81	0.45
4-Hydroxycoumarin_1	neg	7.23	161.02	Level 2	Environmental	foods and additives	Yes	117.41	0.00	206.50	0.14	368.66	0.07	57.11	0.79
Indolepropionic acid	neg	9.23	188.07	Level 2	Endogenous	other endogenous metabolites	Yes	-228.59	0.24	1053.03	0.14	1362.25	0.19	321.14	0.77
3-[(4-chlorobenzoyl)amino]propanoic acid	neg	9.23	226.03	Level 2	Environmental	ambiguous xenobiotic	Yes	-7.79	0.62	86.62	0.14	136.05	0.11	86.73	0.34
Taurolithocholate_2	pos	13.18	466.30	Level 2	Endogenous	bile acids	Yes	8.93	0.72	-130.95	0.15	-174.08	0.18	55.88	0.69
cortisone	neg	11.73	359.19	Level 2	Endogenous	hormones and metabolites	Yes	-2.01	0.78	38.28	0.16	24.46	0.53	-46.92	0.26
N-(2-Propynyl)-N-benzylformamide	pos	11.24	174.09	Level 2	Environmental	ambiguous xenobiotic	Yes	18.89	0.00	-27.82	0.17	-40.63	0.16	-14.60	0.66
Dicyclohexyl phthalate_2	neg	11.78	329.18	Level 2	Environmental	plasticizers and metabolites	Yes	0.59	0.98	113.25	0.17	43.69	0.71	-166.44	0.19
4-METHYL-2-OXO-PENTANOIC ACID	neg	4.58	129.06	Level 2	Endogenous	fatty acids and derivatives	Yes	-37.31	0.87	1118.67	0.17	872.62	0.46	337.17	0.80
Decanoyl-L-Carnitine	pos	14.14	316.25	Level 2	Endogenous	fatty acids and derivatives	Yes	-201.78	0.23	-819.54	0.18	-1115.98	0.20	276.75	0.77
Isoferulic acid 3-O-glucuronide	neg	4.96	369.08	Level 2	Environmental	foods and additives	Yes	53.87	0.00	-66.20	0.18	-98.28	0.16	90.58	0.26
5-hydroxyindoleacetate	pos	4.66	192.07	Level 2	Endogenous	hormones and metabolites	Yes	19.40	0.36	-104.58	0.18	-151.77	0.17	129.98	0.28
alpha-Pinene	pos	14.03	137.13	Level 2	Environmental	plant metabolites and natural products	Yes	-67.89	0.08	-183.67	0.19	-154.83	0.44	-208.22	0.34
N-(9-oxodecyl)acetamide	pos	12.58	214.18	Level 2	Environmental	industrial chemicals and metabolites	Yes	11.61	0.85	-289.80	0.20	-168.71	0.60	-574.10	0.10
17-Methyltestosterone_2	pos	13.78	303.23	Level 2	Endogenous	hormones and metabolites	Yes	12.31	0.21	44.04	0.20	69.20	0.16	7.59	0.89
Dehydroepiandrosterone_1	pos	12.75	289.22	Level 2	Endogenous	hormones and metabolites	Yes	57.11	0.00	97.45	0.21	210.21	0.06	72.90	0.53
Cortodoxone	pos	13.06	329.21	Level 2	Endogenous	hormones and metabolites	Yes	-1.00	0.89	31.97	0.21	24.76	0.50	4.97	0.90
L-Tryptophan	pos	5.15	205.10	Level 2	Endogenous	aminoacids and derivatives	Yes	57.57	0.06	143.68	0.21	237.57	0.15	-62.27	0.72
h-52-drostanolone-m	pos	18.20	287.24	Level 2	Environmental	drugs and metabolites	Yes	3.51	0.94	-219.87	0.21	-197.56	0.43	-242.66	0.35
Butylparaben	neg	9.87	193.09	Level 2	Environmental	personal care chemicals and metabolites	Yes	52.04	0.08	138.62	0.22	319.63	0.05	-418.97	0.02
DL-Coniine	pos	3.32	128.14	Level 2	Environmental	plant metabolites and natural products	Yes	-78.26	0.59	-686.61	0.22	-1061.35	0.19	1670.24	0.06
Bis(2-ethylhexyl)-phthalate	pos	14.21	391.28	Level 2	Environmental	plasticizers and metabolites	Yes	17.13	0.44	96.60	0.23	210.44	0.07	61.75	0.62
FA 18	neg	15.71	311.22	Level 2	Endogenous	fatty acids and derivatives	Yes	207.43	0.10	-538.29	0.23	-938.70	0.15	787.50	0.27
7alpha-hydroxycholest-4-en-3-one	pos	18.90	401.34	Level 2	Endogenous	sterol and phospholipids and derivatives	Yes	-55.37	0.58	-457.19	0.24	-524.84	0.35	84.96	0.88
Digitoxigenin_1	pos	17.59	375.25	Level 2	Environmental	foods and additives	Yes	78.97	0.00	-112.94	0.24	-161.81	0.24	-349.52	0.02
falcarindiol	pos	12.82	225.16	Level 2	Environmental	foods and additives	Yes	82.89	0.00	72.25	0.25	152.65	0.09	-1.29	0.99
3-Methyladipic acid	neg	5.21	159.07	Level 2	Endogenous	fatty acids and derivatives	Yes	23.96	0.41	127.34	0.25	130.74	0.41	-108.74	0.53
Chenodeoxycholic acid_2	pos	16.16	375.29	Level 2	Endogenous	bile acids	Yes	146.07	0.01	243.86	0.26	281.48	0.36	-159.93	0.63
ferulic acid	neg	6.68	193.05	Level 2	Environmental	foods and additives	Yes	84.62	0.02	-143.12	0.26	-322.30	0.07	104.38	0.59
ethyl-3-indoleacetate	pos	12.52	204.10	Level 2	Environmental	foods and additives	Yes	-22.14	0.13	58.75	0.27	76.93	0.31	80.59	0.32
2-Hydroxyhippuric acid	neg	5.82	194.04	Level 2	Environmental	personal care chemicals and metabolites	Yes	416.23	0.07	-975.73	0.27	-2140.75	0.09	2175.03	0.10
4'-ethenyl-2'-hydroxy-1,4',4a-trimethyl-5-oxospiro[2,3,4,7,8,8a-hexahydronaphthalene-6,1'-cyclopentane]-1-carboxylic acid	pos	14.70	335.22	Level 2	Ambigous	ambiguous	Yes	21.30	0.46	-111.34	0.27	-167.74	0.25	94.20	0.55
14-(hydroxymethyl)-5,9-dimethyl-15-oxapentacyclo[11.3.1.0,?.0?,?.0?,?]heptadecane-5-carboxylic acid_1	neg	12.85	333.21	Level 2	Environmental	plant metabolites and natural products	Yes	1678.56	0.00	1416.21	0.28	3191.25	0.09	210.93	0.91
humulone_1	pos	16.14	363.22	Level 2	Environmental	foods and additives	Yes	-114.46	0.02	187.61	0.28	248.64	0.32	-145.81	0.60
Cyclopamine	pos	15.21	412.32	Level 2	Environmental	plant metabolites and natural products	Yes	7.59	0.38	-33.37	0.29	-5.40	0.91	-46.05	0.37
Oleic acid_1	neg	18.49	281.25	Level 2	Endogenous	fatty acids and derivatives	Yes	-3684.05	0.01	-5321.62	0.29	1694.37	0.82	-5021.60	0.52
O-dodecanoylcarnitine	pos	15.72	344.28	Level 2	Endogenous	fatty acids and derivatives	Yes	-29.71	0.73	-316.16	0.30	-431.31	0.32	-70.49	0.88
Dehydroepiandrosterone_2	pos	12.81	271.21	Level 2	Endogenous	hormones and metabolites	Yes	597.17	0.00	479.18	0.30	1112.58	0.09	48.90	0.94
Triethylene glycol monomethyl ether	pos	5.44	165.11	Level 2	Environmental	industrial chemicals and metabolites	Yes	-69.43	0.07	-145.93	0.30	-112.14	0.58	308.24	0.16

2-Amino-1-phenylethanol_2	pos	7.08	120.08	Level 2	Endogenous	other endogenous metabolites	Yes	53.27	0.00	-41.29	0.31	-86.56	0.14	87.84	0.16
Octanoyl-L-Carnitine	pos	11.81	288.22	Level 2	Endogenous	fatty acids and derivatives	Yes	-107.42	0.29	-375.17	0.31	-439.31	0.41	427.43	0.45
3-Carboxy-4-methyl-5-propyl-2-furanpropionic acid (CMPF)	pos	11.94	241.11	Level 2	Endogenous	fatty acids and derivatives	Yes	611.62	0.00	676.74	0.31	1098.41	0.25	-2205.78	0.03
2-(2-hydroxybut-3-en-2-yl)-3a,6,6,9a-tetramethyl-2,4,5,5a,7,8,9,9b-octahydro-1H-benzo[e][1]benzofuran-4,5-diol	pos	15.63	356.28	Level 2	Environmental	plant metabolites and natural products	Yes	26.53	0.63	-204.75	0.31	-143.70	0.62	166.36	0.58
(1S,5R,9S,13R)-14-formyl-5,9-dimethyltetracyclo[11.2.1.0,?.0?,?]hexadec-14-ene-5-carboxylic acid	pos	12.85	317.21	Level 2	Ambigous	ambiguous	Yes	763.76	0.00	600.39	0.31	1487.70	0.08	-58.48	0.95
Metoprolol	pos	8.91	268.19	Level 2	Environmental	drugs and metabolites	Yes	114.36	0.18	-480.17	0.32	-694.53	0.31	-350.10	0.63
Benzoylcholine	pos	12.65	208.13	Level 2	Environmental	plant metabolites and natural products	Yes	-4.74	0.85	-99.42	0.32	-135.28	0.34	197.17	0.20
cis-4,10,13,16-Docosatetraenoic acid	neg	18.52	331.26	Level 2	Endogenous	fatty acids and derivatives	Yes	-192.30	0.05	-364.43	0.32	228.85	0.66	-569.17	0.31
4-Hydroxybenzenesulfonic acid	neg	4.73	172.99	Level 2	Environmental	industrial chemicals and metabolites	Yes	-80.85	0.80	-1157.95	0.33	-2564.11	0.13	280.40	0.88
N-Desmethyltramadol	pos	11.92	250.18	Level 2	Environmental	drugs and metabolites	Yes	-6.87	0.43	31.92	0.34	56.10	0.24	33.41	0.51
Hesperetin	pos	9.31	303.09	Level 2	Environmental	foods and additives	Yes	41.62	0.19	-104.45	0.34	-157.55	0.32	88.47	0.60
Androsterone sulfate	neg	13.90	369.17	Level 2	Endogenous	hormones and metabolites	Yes	-130.71	0.17	-352.51	0.35	-63.42	0.91	1049.50	0.07
pregnenolone sulfate	neg	13.52	395.19	Level 2	Endogenous	sterol and phospholipids and derivatives	Yes	-88.92	0.07	-173.70	0.36	-138.74	0.61	435.76	0.13
1-Hydroxy-2-naphthoic acid	neg	10.10	187.04	Level 2	Environmental	industrial chemicals and metabolites	Yes	11.47	0.25	-32.54	0.37	-20.45	0.69	24.85	0.69
Phthalic anhydride	pos	6.66	149.02	Level 2	Environmental	industrial chemicals and metabolites	Yes	-1.64	0.98	-237.75	0.38	-658.11	0.09	-125.76	0.77
sebacic acid	neg	9.93	201.11	Level 2	Endogenous	fatty acids and derivatives	Yes	87.75	0.19	219.92	0.38	-12.79	0.97	162.12	0.67
azelaic acid	neg	8.52	187.10	Level 2	Environmental	personal care chemicals and metabolites	Yes	157.39	0.00	-161.61	0.38	-242.24	0.36	502.82	0.08
3,4-Dimethoxycinnamic acid	pos	8.32	209.08	Level 2	Environmental	foods and additives	Yes	147.89	0.00	-130.09	0.39	-232.41	0.28	408.86	0.09
1,7-dimethyluric acid	neg	5.38	195.05	Level 2	Environmental	foods and additives	Yes	298.86	0.00	-261.83	0.39	-642.48	0.13	186.37	0.69
Retinol	pos	18.23	269.23	Level 2	Environmental	foods and additives	Yes	-2.34	0.99	-588.93	0.40	-662.58	0.51	-685.21	0.50
4-Pregnene-11 Corticosterone	pos	12.07	347.22	Level 2	Endogenous	hormones and metabolites	Yes	-4.32	0.72	36.77	0.40	49.36	0.43	-16.91	0.80
1H-2-benzopyran-1-one, 6,8-dihydroxy-3-methyl-	neg	11.39	191.03	Level 2	Ambigous	ambiguous	Yes	4.41	0.69	-34.69	0.40	-76.44	0.19	-112.97	0.08
14-(hydroxymethyl)-5,9-dimethyl-15-oxapentacyclo[11.3.1.0,?.0?,?.0?,?]heptadecane-5-carboxylic acid_2	neg	15.32	333.21	Level 2	Environmental	plant metabolites and natural products	Yes	175.71	0.00	162.34	0.41	478.39	0.09	435.89	0.15
vitamin D3	pos	16.12	385.35	Level 2	Endogenous	other endogenous metabolites	Yes	5.32	0.38	-19.15	0.41	-30.46	0.36	-51.17	0.16
9-hydroxy-7-(2-hydroxypropan-2-yl)-1,4a-dimethyl-2,3,4,9,10,10a-hexahydrophenanthrene-1-carboxylic acid	neg	13.87	331.19	Level 2	Ambigous	ambiguous	Yes	178.90	0.00	190.98	0.41	485.36	0.14	-144.55	0.69
Quinoline_3	pos	5.99	130.07	Level 2	Environmental	industrial chemicals and metabolites	Yes	-47.11	0.03	-69.35	0.41	-15.05	0.90	126.64	0.33
a-Linolenic acid	neg	17.57	277.22	Level 2	Endogenous	fatty acids and derivatives	Yes	-742.39	0.12	-1362.97	0.42	464.54	0.85	-3430.89	0.19
5-[(8aS)-2,5,5,8a-tetramethyl-3-oxo-4a,6,7,8-tetrahydro-4H-naphthalen-1-yl]-3-methylpentanoic acid_2	pos	16.30	343.22	Level 2	Ambigous	ambiguous	Yes	-59.88	0.38	-189.79	0.42	-2.19	0.99	-569.39	0.12
myrcene_1	pos	9.30	159.12	Level 2	Environmental	foods and additives	Yes	-10.79	0.76	99.18	0.42	30.90	0.86	116.12	0.54
Linoleic acid_1	pos	16.37	281.25	Level 2	Endogenous	fatty acids and derivatives	Yes	-115.53	0.13	-208.13	0.43	-6.05	0.99	-238.71	0.56
Cholic Acid_3	pos	13.78	373.27	Level 2	Endogenous	bile acids	Yes	3.72	0.83	-49.73	0.44	-120.55	0.19	-106.59	0.29
Ephedrine	pos	6.44	166.12	Level 2	Environmental	drugs and metabolites	Yes	-4.33	0.71	34.73	0.45	25.32	0.70	40.14	0.56
N-Acetylcytidine	pos	4.72	286.10	Level 2	Endogenous	other endogenous metabolites	Yes	-12.30	0.40	-42.20	0.45	-93.55	0.24	55.19	0.52
Hexanoyl-L-Carnitine	pos	8.26	260.19	Level 2	Endogenous	fatty acids and derivatives	Yes	-14.64	0.73	-117.81	0.46	-140.36	0.54	96.00	0.69
Monoolein	pos	18.42	357.30	Level 2	Endogenous	fatty acids and derivatives	Yes	366.16	0.08	-575.04	0.46	33.46	0.98	-1836.34	0.13
Indole-3-acetyl-L-glutamic acid	pos	7.59	305.11	Level 2	Endogenous	aminoacids and derivatives	Yes	-5.22	0.48	-20.68	0.46	-13.47	0.74	47.38	0.27
4-hydroxybenzaldehyde	pos	4.90	105.03	Level 2	Environmental	plant metabolites and natural products	Yes	1048.73	0.00	690.07	0.46	868.83	0.52	3235.58	0.03
Palmitoyl-carnitine	pos	17.73	400.34	Level 2	Endogenous	fatty acids and derivatives	Yes	39.31	0.66	251.74	0.47	790.90	0.11	-128.18	0.80
Chenodeoxycholic acid_3	pos	16.16	785.59	Level 2	Endogenous	bile acids	Yes	175.76	0.00	118.03	0.47	211.01	0.37	-292.40	0.26
O-Arachidonoylglycidol	pos	17.38	361.27	Level 2	Endogenous	fatty acids and derivatives	Yes	9.43	0.57	43.62	0.48	67.02	0.44	85.71	0.36
5-(4-carboxy-3-methylbutyl)-5,6,8a-trimethyl-3-oxo-4a,6,7,8-tetrahydro-4H-naphthalene-1-carboxylic acid	pos	8.19	373.20	Level 2	Ambigous	ambiguous	Yes	25.21	0.00	15.46	0.48	15.67	0.62	36.37	0.29
6,8-Dimethyl-4-hydroxycoumarin	neg	10.90	189.06	Level 2	Environmental	plant metabolites and natural products	Yes	97.37	0.01	97.55	0.49	210.45	0.30	7.38	0.97
3-Dehydrocholic acid	neg	13.70	405.26	Level 2	Endogenous	bile acids	Yes	50.47	0.08	-71.05	0.50	-127.65	0.40	-110.93	0.50
bergenin	pos	11.92	329.09	Level 2	Environmental	plant metabolites and natural products	Yes	11.45	0.09	16.82	0.50	23.28	0.52	-42.65	0.27
Eupatilin	neg	9.66	343.09	Level 2	Environmental	drugs and metabolites	Yes	7.44	0.44	-23.89	0.50	-45.96	0.37	-42.28	0.44
Glycocholic acid_2	pos	13.77	448.31	Level 2	Endogenous	bile acids	Yes	90.59	0.11	134.45	0.51	229.38	0.43	-62.94	0.84
deoxycholate	pos	15.03	357.28	Level 2	Endogenous	bile acids	Yes	68.07	0.16	117.84	0.51	158.15	0.54	-196.54	0.48
Theophyline	neg	6.03	179.06	Level 2	Environmental	foods and additives	Yes	818.81	0.02	841.29	0.51	315.48	0.86	-758.82	0.70
glycodeoxycholic acid_2	pos	13.14	430.30	Level 2	Endogenous	bile acids	Yes	-7.26	0.48	-24.29	0.51	-34.45	0.52	-62.39	0.27
2-Aminoacetophenone	pos	9.08	136.08	Level 2	Environmental	foods and additives	Yes	60.97	0.01	-60.04	0.52	-129.44	0.33	130.68	0.35
Sedoheptulose 7-phosphate	neg	7.40	289.04	Level 2	Endogenous	other endogenous metabolites	Yes	26.88	0.01	23.85	0.52	12.44	0.82	-31.73	0.58
N-Benzyl-9-(tetrahydro-2H-pyran-2-yl)adenine	pos	10.65	310.16	Level 2	Endogenous	other endogenous metabolites	Yes	7.48	0.26	15.57	0.53	52.47	0.14	-1.73	0.96
Amantadine	pos	10.22	152.14	Level 2	Environmental	drugs and metabolites	Yes	-4.55	0.63	20.65	0.53	57.34	0.22	25.13	0.62
Monolinolenin	pos	17.59	353.27	Level 2	Endogenous	fatty acids and derivatives	Yes	152.66	0.02	-148.61	0.54	-35.19	0.92	-510.34	0.17
Conjugated linoleic Acid	pos	18.00	263.24	Level 2	Environmental	foods and additives	Yes	193.48	0.18	-319.71	0.54	18.89	0.98	-1195.41	0.14
P-cresol sulfate	neg	6.98	187.01	Level 2	Endogenous	other endogenous metabolites	Yes	471.16	0.48	1510.65	0.55	1179.65	0.74	3502.82	0.36
5-[(8aS)-2,5,5,8a-tetramethyl-3-oxo-4a,6,7,8-tetrahydro-4H-naphthalen-1-yl]-3-methylpentanoic acid_1	pos	16.60	338.27	Level 2	Ambigous	ambiguous	Yes	-21.46	0.41	-54.20	0.55	35.11	0.79	-200.97	0.16
Linoleyl-carnitine	pos	17.41	424.34	Level 2	Endogenous	fatty acids and derivatives	Yes	-20.52	0.69	113.40	0.56	394.39	0.15	-339.52	0.25
3-(3-hydroxyphenyl)propionic acid	neg	6.81	165.06	Level 2	Environmental	foods and additives	Yes	168.99	0.39	-412.75	0.56	-603.59	0.55	1594.38	0.15
3,5-Dimethoxycinnamic acid	neg	10.73	207.07	Level 2	Environmental	foods and additives	Yes	12.62	0.72	-76.46	0.56	-321.67	0.09	-155.00	0.45
Cyclo(Leu-Pro)	pos	8.55	211.14	Level 2	Endogenous	aminoacids and derivatives	Yes	569.89	0.00	395.01	0.57	291.90	0.77	39.02	0.97
cyclo(L-Val-L-Pro)	pos	6.92	197.13	Level 2	Endogenous	aminoacids and derivatives	Yes	135.03	0.29	271.40	0.57	633.27	0.35	-376.66	0.61
9-HOTrE	neg	15.62	293.21	Level 2	Endogenous	fatty acids and derivatives	Yes	77.94	0.59	-283.17	0.57	-37.95	0.96	-1074.93	0.16
4-Hydroxymandelonitrile	pos	9.47	150.06	Level 2	Endogenous	other endogenous metabolites	Yes	27.37	0.00	16.26	0.57	-14.54	0.72	75.61	0.09
Octanedioic acid	neg	6.88	173.08	Level 2	Environmental	industrial chemicals and metabolites	Yes	33.56	0.39	81.37	0.58	21.51	0.92	267.36	0.24
D-sphingosine	pos	18.04	300.29	Level 2	Endogenous	sterol and phospholipids and derivatives	Yes	-101.91	0.17	150.33	0.58	744.63	0.05	-412.59	0.32
2,4,5-Trimethoxybenzaldehyde	neg	8.02	195.07	Level 2	Environmental	plant metabolites and natural products	Yes	45.88	0.10	-53.56	0.58	-171.36	0.22	-74.05	0.62
(2Z,6E,10E)-12-hydroxy-6,10-dimethyl-2-(4-methylpent-3-enyl)dodeca-2,6,10-trienoic acid	pos	16.23	303.23	Level 2	Environmental	plant metabolites and natural products	Yes	-104.98	0.61	-385.00	0.58	229.21	0.82	-1237.38	0.26

Glycochenodeoxycholic acid_2	pos	13.25	414.30	Level 2	Endogenous	bile acids	Yes	25.62	0.56	-86.43	0.59	-151.72	0.50	-96.82	0.69
Indole-3-acetic acid	pos	8.47	176.07	Level 2	Endogenous	other endogenous metabolites	Yes	28.49	0.51	-82.27	0.59	-11.95	0.96	-183.68	0.43
Bufalin	pos	13.71	387.25	Level 2	Environmental	plant metabolites and natural products	Yes	13.15	0.70	78.78	0.59	66.21	0.75	-6.40	0.97
indole-3-acetate	pos	7.53	176.07	Level 2	Endogenous	other endogenous metabolites	Yes	359.45	0.06	-391.58	0.59	-794.10	0.44	1288.34	0.24
Myristoyl-carnitine_1	pos	16.86	372.31	Level 2	Endogenous	fatty acids and derivatives	Yes	61.53	0.25	-107.28	0.59	31.86	0.91	52.99	0.86
p-Coumaraldehyde_2	pos	10.64	149.06	Level 2	Environmental	foods and additives	Yes	-120.01	0.17	-165.39	0.60	-277.50	0.54	354.29	0.48
4-Toluenesulfonamide	pos	10.21	172.04	Level 2	Environmental	drugs and metabolites	Yes	16.79	0.57	61.93	0.60	130.18	0.44	51.86	0.77
9Z, 11E-Linoleic acid	neg	18.00	279.23	Level 2	Endogenous	fatty acids and derivatives	Yes	-2293.82	0.03	-1925.54	0.60	3339.20	0.53	-6075.59	0.29
Taurolithocholate_1	neg	15.48	482.30	Level 2	Endogenous	bile acids	Yes	11.93	0.40	-25.46	0.61	-57.71	0.41	46.54	0.54
Linoleic acid_4	pos	14.59	303.23	Level 2	Endogenous	fatty acids and derivatives	Yes	10.80	0.65	42.15	0.62	43.47	0.72	92.58	0.51
Chenodeoxycholic acid_1	pos	13.37	357.28	Level 2	Endogenous	bile acids	Yes	20.51	0.46	-50.66	0.62	7.98	0.96	144.36	0.36
3,5-Dibromo-4-hydroxybenzoic acid	neg	11.84	292.85	Level 2	Environmental	pesticides and metabolites	Yes	-40.71	0.09	-40.45	0.62	-30.62	0.80	166.40	0.19
Tramadol	pos	8.79	264.20	Level 2	Environmental	drugs and metabolites	Yes	-32.83	0.63	-343.65	0.63	-718.35	0.48	-2306.08	0.03
humulone_2	pos	13.73	385.20	Level 2	Environmental	foods and additives	Yes	25.03	0.01	17.70	0.63	49.05	0.35	-54.64	0.34
Tauroallocholic acid	neg	13.83	514.28	Level 2	Endogenous	bile acids	Yes	-26.06	0.59	-83.08	0.63	-310.83	0.21	142.90	0.60
Tridecanedioic acid	neg	13.08	243.16	Level 2	Endogenous	fatty acids and derivatives	Yes	21.56	0.72	105.10	0.64	87.99	0.78	-61.82	0.86
Cholest-4,6-Dien-3-One	pos	16.14	383.33	Level 2	Endogenous	sterol and phospholipids and derivatives	Yes	-4.72	0.59	-15.20	0.64	-12.99	0.78	27.47	0.58
(3,5-dimethoxyphenyl)methan-1-ol	pos	11.39	151.08	Level 2	Environmental	foods and additives	Yes	-15.54	0.87	151.64	0.64	417.13	0.37	102.52	0.84
Traumatic Acid	neg	11.42	227.13	Level 2	Environmental	plant metabolites and natural products	Yes	45.96	0.37	82.92	0.65	83.62	0.75	-183.44	0.52
Phenylalanylphenylalanine	pos	9.26	313.15	Level 2	Endogenous	aminoacids and derivatives	Yes	-68.53	0.18	-84.21	0.65	-248.60	0.35	253.91	0.37
Indole-3-acetamide	pos	7.62	175.09	Level 2	Environmental	foods and additives	Yes	9.27	0.92	143.68	0.65	9.09	0.98	307.71	0.54
Indole-3-acetyl-L-alanine	pos	7.70	247.11	Level 2	Endogenous	aminoacids and derivatives	Yes	16.37	0.35	28.95	0.65	60.31	0.51	17.10	0.86
Tropic acid	neg	5.53	165.05	Level 2	Environmental	industrial chemicals and metabolites	Yes	135.18	0.00	72.85	0.67	15.78	0.95	23.61	0.93
9-HODE	neg	16.03	295.23	Level 2	Endogenous	fatty acids and derivatives	Yes	67.58	0.82	-430.94	0.67	315.17	0.83	-1970.70	0.21
Solasodin	pos	13.99	414.34	Level 2	Environmental	plant metabolites and natural products	Yes	121.65	0.02	78.93	0.67	295.89	0.26	-100.50	0.73
16-HDoHE	neg	16.16	343.23	Level 2	Endogenous	fatty acids and derivatives	Yes	28.31	0.90	-319.80	0.67	341.73	0.75	-1705.19	0.14
Testosterone propionate	pos	16.52	345.24	Level 2	Environmental	drugs and metabolites	Yes	-16.64	0.62	-48.95	0.67	79.86	0.63	-345.60	0.05
2-methylhippuric acid_1	pos	12.59	194.08	Level 2	Environmental	industrial chemicals and metabolites	Yes	18.50	0.02	-12.09	0.68	8.31	0.84	36.04	0.42
3-Methyloxyindole	pos	10.22	148.08	Level 2	Environmental	foods and additives	Yes	97.22	0.04	-77.24	0.68	-106.15	0.69	129.63	0.65
Phenylacetylglutamine	pos	5.60	265.12	Level 2	Endogenous	aminoacids and derivatives	Yes	263.77	0.28	-365.20	0.69	-966.20	0.46	2936.84	0.04
Cyclamate	neg	5.58	178.05	Level 2	Environmental	foods and additives	Yes	53.55	0.36	93.50	0.69	-282.02	0.40	-282.57	0.38
2-Amino-3-hydroxy-3-phenylpropanoic acid	pos	9.13	164.07	Level 2	Environmental	foods and additives	Yes	44.63	0.01	-26.63	0.69	-51.33	0.59	124.80	0.21
Hippuric acid	neg	4.89	178.05	Level 2	Endogenous	other endogenous metabolites	Yes	1527.94	0.00	441.84	0.69	-18.13	0.99	3684.62	0.04
glycolithocholic acid_2	pos	13.35	434.33	Level 2	Endogenous	bile acids	Yes	9.49	0.26	12.23	0.69	36.26	0.41	-14.34	0.76
Sulfochenodeoxycholic acid	neg	13.63	471.24	Level 2	Endogenous	bile acids	Yes	30.83	0.18	33.20	0.70	25.79	0.83	-80.82	0.53
Gentisic acid	neg	2.35	153.02	Level 2	Endogenous	other endogenous metabolites	Yes	53.94	0.11	-48.53	0.70	-204.26	0.26	252.77	0.19
inosine	neg	2.85	267.07	Level 2	Endogenous	other endogenous metabolites	Yes	-8.81	0.73	34.62	0.70	-32.05	0.80	90.40	0.52
1-methylxanthine	pos	4.29	167.06	Level 2	Environmental	foods and additives	Yes	263.10	0.00	122.41	0.70	76.99	0.87	418.27	0.40
Arachidonic acid	neg	17.86	303.23	Level 2	Endogenous	fatty acids and derivatives	Yes	-571.43	0.21	-607.54	0.70	1693.21	0.46	-2839.84	0.26
lenticin	pos	5.90	247.14	Level 2	Environmental	foods and additives	Yes	-295.19	0.05	-206.56	0.70	390.06	0.62	-1152.16	0.17
2-Amino-1-phenylethanol_3	pos	6.46	120.08	Level 2	Endogenous	other endogenous metabolites	Yes	37.08	0.02	-21.58	0.71	-123.31	0.14	52.08	0.55
Myristoyl-carnitine_2	pos	18.01	372.31	Level 2	Endogenous	fatty acids and derivatives	Yes	246.76	0.05	-168.03	0.71	53.48	0.93	-1152.04	0.10
paraxanthine	pos	5.86	181.07	Level 2	Environmental	foods and additives	Yes	2308.99	0.00	1004.24	0.73	-2.41	1.00	2468.86	0.59
Phe-Trp	pos	8.92	352.17	Level 2	Endogenous	aminoacids and derivatives	Yes	-39.39	0.47	65.36	0.73	-75.60	0.78	75.58	0.80
alpha-Linolenoyl ethanolamide	pos	17.84	322.27	Level 2	Endogenous	fatty acids and derivatives	Yes	-80.13	0.36	-103.85	0.74	411.24	0.36	-556.97	0.26
3,4-dimethoxyphenylacetic acid	neg	6.99	195.07	Level 2	Environmental	foods and additives	Yes	172.31	0.16	-138.28	0.75	-545.13	0.38	818.18	0.23
Glycyrrhetinic acid	pos	7.74	471.35	Level 2	Environmental	foods and additives	Yes	-6.88	0.86	-44.26	0.76	-1.94	0.99	147.35	0.49
Undecanedioic acid	neg	11.13	215.13	Level 2	Endogenous	fatty acids and derivatives	Yes	10.77	0.77	41.36	0.76	45.04	0.82	168.25	0.43
Methyl indole-3-acetate	pos	11.52	190.09	Level 2	Endogenous	other endogenous metabolites	Yes	-12.76	0.73	-41.05	0.76	-122.53	0.53	199.35	0.35
2-methylhippuric acid_2	pos	14.20	194.08	Level 2	Environmental	industrial chemicals and metabolites	Yes	16.45	0.16	12.87	0.77	-8.48	0.89	64.14	0.34
3-[N-(2-indol-3-ylethyl)carbamoyl]propanoic acid	pos	8.89	283.11	Level 2	Environmental	plant metabolites and natural products	Yes	-4.82	0.76	16.24	0.77	3.07	0.97	29.99	0.73
Indole-3-butyric acid	pos	10.46	204.10	Level 2	Environmental	foods and additives	Yes	1.47	0.95	-23.59	0.78	-94.95	0.43	126.08	0.33
2-Amino-1-phenylethanol_1	pos	9.02	120.08	Level 2	Endogenous	other endogenous metabolites	Yes	-5.47	0.75	17.70	0.78	-40.09	0.66	48.10	0.61
Bilirubin	pos	16.96	585.27	Level 2	Endogenous	other endogenous metabolites	Yes	-27.87	0.13	19.26	0.78	86.39	0.38	-10.07	0.92
(R)-4-((8S,9S,10R,13R,14S,17R)-10,13-dimethyl-3-oxo-2,3,6,7,8,9,10,11,12,13,14,15,16,17-tetradecahydro-1H-cyclopenta[a]phenanthren-17-yl)pentanoic acid	pos	13.98	337.25	Level 2	Endogenous	bile acids	Yes	0.55	0.98	-17.48	0.79	-95.76	0.31	17.57	0.87
SodiuM-taurodeoxycholate	neg	14.69	498.29	Level 2	Endogenous	bile acids	Yes	-28.26	0.76	87.08	0.79	-162.09	0.74	-55.86	0.91
Dodecanedioic acid	neg	12.19	229.14	Level 2	Endogenous	fatty acids and derivatives	Yes	223.38	0.02	86.85	0.80	-342.62	0.49	383.98	0.48
3beta,5beta-tetrahydrocortisone	pos	10.66	365.23	Level 2	Endogenous	hormones and metabolites	Yes	7.77	0.32	-7.01	0.81	-12.50	0.77	47.80	0.29
1-(9Z-Octadecenoyl)-sn-glycero-3-phospho-(1'-myo-inositol)	neg	16.98	597.30	Level 2	Endogenous	sterol and phospholipids and derivatives	Yes	8.92	0.27	-6.88	0.82	-14.67	0.73	-23.85	0.60
Hydroxycotinine	pos	5.24	193.10	Level 2	Endogenous	hormones and metabolites	Yes	232.73	0.26	-170.95	0.82	-16.39	0.99	-392.58	0.79
Avocadyne Acetate	neg	17.98	325.24	Level 2	Endogenous	fatty acids and derivatives	Yes	-19.27	0.52	-23.73	0.83	65.30	0.67	113.49	0.49
1-naphthalenesulfonate	neg	8.08	207.01	Level 2	Environmental	industrial chemicals and metabolites	Yes	-21.64	0.46	24.68	0.83	-4.22	0.98	127.60	0.45
Adenosine	pos	4.93	268.10	Level 2	Endogenous	other endogenous metabolites	Yes	-103.47	0.20	61.58	0.83	-51.76	0.90	77.28	0.86
Saccharin	neg	4.41	181.99	Level 2	Environmental	foods and additives	Yes	57.00	0.35	44.60	0.84	-356.46	0.27	-302.45	0.36
glycolithocholic acid_1	neg	15.64	432.31	Level 2	Endogenous	bile acids	Yes	83.01	0.03	26.41	0.84	68.20	0.72	180.80	0.38
(6E)-2,6,10-trimethyldodeca-6,11-diene-2,3,10-triol	neg	17.71	255.20	Level 2	Ambigous	ambiguous	Yes	9.42	0.80	-26.62	0.84	-24.00	0.90	180.78	0.39
2-Hydroxyquinoline	pos	8.01	146.06	Level 2	Environmental	other contaminants and metabolites	Yes	481.02	0.02	-146.66	0.85	-380.55	0.74	89.14	0.94
Glycocholic acid_1	neg	13.96	464.30	Level 2	Endogenous	bile acids	Yes	19.70	0.71	-34.00	0.86	-250.88	0.36	220.11	0.45
N-Tetracosenoyl-4-sphingenine	pos	17.65	282.28	Level 2	Endogenous	sterol and phospholipids and derivatives	Yes	-37.95	0.56	-32.77	0.88	363.61	0.26	-521.94	0.14
Stercobilin	pos	11.82	595.35	Level 2	Endogenous	other endogenous metabolites	Yes	68.95	0.04	17.14	0.89	184.50	0.29	-21.92	0.91
Isotretinoin	pos	16.06	301.22	Level 2	Environmental	drugs and metabolites	Yes	70.56	0.45	-42.42	0.90	88.21	0.85	-941.55	0.07
Meloxicam	pos	10.47	352.04	Level 2	Environmental	drugs and metabolites	Yes	5.15	0.09	1.41	0.90	-4.34	0.78	-16.79	0.32
(2R)-2-[(2R,5S)-5-[(2S)-2-hydroxybutyl]oxolan-2-yl]propanoic acid	pos	11.15	217.14	Level 2	Ambigous	ambiguous	Yes	-87.64	0.09	-24.19	0.90	21.92	0.94	-180.19	0.53
Cholic Acid_1	neg	15.17	407.28	Level 2	Endogenous	bile acids	Yes	334.26	0.02	-66.11	0.90	-279.03	0.70	-339.59	0.67

16-hydroxypalmitic acid	neg	16.79	271.23	Level 2	Endogenous	fatty acids and derivatives	Yes	-189.62	0.09	-47.96	0.90	507.57	0.36	-14.39	0.98
3-Indoxyl sulfate	neg	5.08	212.00	Level 2	Endogenous	other endogenous metabolites	Yes	59.52	0.76	-84.76	0.91	-57.99	0.96	2377.14	0.03
Dicyclohexyl phthalate_1	pos	12.56	331.19	Level 2	Environmental	plasticizers and metabolites	Yes	214.49	0.06	-40.86	0.92	365.54	0.53	163.55	0.80
Glycoursodeoxycholic acid	neg	14.81	448.31	Level 2	Endogenous	bile acids	Yes	193.52	0.16	-37.46	0.94	-207.16	0.77	-315.56	0.67
Alpha-Hydroxydeoxycholic Acid	neg	15.00	391.29	Level 2	Endogenous	bile acids	Yes	129.64	0.16	-24.41	0.94	-352.51	0.46	-900.74	0.08
DL-Phenylalanine	pos	6.48	166.09	Level 2	Endogenous	aminoacids and derivatives	Yes	68.57	0.00	5.96	0.94	-86.51	0.48	203.90	0.11
4-tert-Butylcatechol	neg	12.56	165.09	Level 2	Environmental	industrial chemicals and metabolites	Yes	-22.50	0.87	-33.58	0.95	385.50	0.58	-174.48	0.82
Diosgenin	pos	15.26	415.32	Level 2	Environmental	plant metabolites and natural products	Yes	-23.25	0.18	-4.16	0.95	77.58	0.41	-36.40	0.72
2,6-dihydroxybenzoic acid	neg	6.03	153.02	Level 2	Environmental	foods and additives	Yes	153.24	0.09	20.03	0.95	10.26	0.98	81.82	0.88
4-Methoxycinnamic acid 1	neg	9.52	177.06	Level 2	Environmental	foods and additives	Yes	-21.24	0.61	-8.34	0.96	-203.24	0.36	120.23	0.61
Tauroursodeoxycholic acid_1	pos	14.68	464.28	Level 2	Endogenous	bile acids	Yes	-21.82	0.78	14.06	0.96	-154.10	0.71	87.46	0.84
valproic acid	neg	13.48	143.11	Level 2	Environmental	drugs and metabolites	Yes	705.44	0.09	-77.38	0.96	-81.14	0.97	4290.78	0.07
Glycochenodeoxycholic acid_1	pos	14.81	432.31	Level 2	Endogenous	bile acids	Yes	104.79	0.16	10.27	0.97	-67.88	0.86	-141.76	0.72
7-ethenyl-1,4a,7-trimethyl-3,4,6,8,8a,9,10,10a-octahydro-2H-phenanthrene-1-carboxylic acid	neg	17.49	301.22	Level 2	Environmental	plant metabolites and natural products	Yes	20.67	0.96	-33.93	0.98	1133.39	0.54	-3158.61	0.13
Theobromine	pos	5.07	181.07	Level 2	Environmental	foods and additives	Yes	-45.42	0.90	34.03	0.98	-1.62	1.00	517.34	0.79
1-Hydroxy-2-(9Z,12Z-octadecadienoyl)-sn-glycero-3-phosphoethanolamine	neg	17.41	476.28	Level 2	Endogenous	sterol and phospholipids and derivatives	Yes	11.97	0.14	0.76	0.98	-2.93	0.95	58.12	0.21
Quinoline_2	pos	11.51	130.07	Level 2	Environmental	industrial chemicals and metabolites	Yes	-6.48	0.89	3.77	0.98	-156.02	0.51	294.18	0.25
Linoleic acid_2	pos	17.52	303.23	Level 2	Endogenous	fatty acids and derivatives	Yes	-5.70	0.94	3.87	0.99	287.55	0.43	-585.50	0.14
p-Coumaraldehyde_1	neg	5.52	147.05	Level 2	Environmental	foods and additives	Yes	29.86	0.00	0.26	0.99	-13.61	0.76	44.98	0.34
methyl (4R)-4-((3R,5S,7R,9S,10S,13R,15R,17R)-3,7,15-trihydroxy-10,13-dimethylhexadecahydro-1H-cyclopenta[a]phenanthren-17-yl)pentanoate	pos	15.46	445.29	Level 2	Endogenous	bile acids	Yes	220.30	0.00	1.77	0.99	76.49	0.85	-25.57	0.95
(5E)-4,9-dihydroxy-6-methyl-3,10-dimethylidene-4,7,8,9,11,11a-hexahydro-3aH-cyclodeca[b]furan-2-one	neg	12.36	263.13	Level 2	Ambigous	ambiguous	No								
(2S,3S,4S,5R,6S)-3,4,5-trihydroxy-6-(5-hydroxy-4-oxo-2-phenylchromen-7-yl)oxyoxane-2-carboxylic acid	neg	7.42	429.08	Level 2	Ambigous	ambiguous	No								
(2S,3S,4S,5R,6R)-6-[[(3S,6aR,6bS,8aS,14bR)-8a-carboxy-4-(hydroxymethyl)-4,6a,6b,11,11,14b-hexamethyl-1,2,3,4a,5,6,7,8,9,10,12,12a,14,14a-tetradecahydropicen-3-yl]oxy]-3,4,5-trihydroxyoxane-2-carboxylic acid	neg	15.56	647.38	Level 2	Ambigous	ambiguous	No								
(1s,4r)-7,7-dimethyl-2-oxobicyclo[2.2.1]heptane-1-carboxylic acid_1	pos	20.96	165.09	Level 2	Ambigous	ambiguous	No								
(1s,4r)-7,7-dimethyl-2-oxobicyclo[2.2.1]heptane-1-carboxylic acid_2	pos	18.73	165.09	Level 2	Ambigous	ambiguous	No								
2,4-dihydroxyheptadecyl acetate	pos	16.54	313.27	Level 2	Ambigous	ambiguous	No								
3,8-di((E)-but-2-en-1-yl)-1-methyl-7,8-dihydro-1H-imidazo[2,1-f]purine-2,4(3H,6H)-dione	pos	10.91	338.16	Level 2	Ambigous	ambiguous	No								
dimethyl 2,4-bis(4-hydroxyphenyl)cyclobutane-1,3-dicarboxylate	pos	13.11	357.13	Level 2	Ambigous	ambiguous	No								
DL-3-Phenyllactic acid	neg	6.85	331.12	Level 2	Endogenous	aminoacids and derivatives	No								
L-Kynurenine	pos	7.75	103.05	Level 2	Endogenous	aminoacids and derivatives	No								
pyroglutamate	pos	1.42	130.05	Level 2	Endogenous	aminoacids and derivatives	No								
L-Tyrosine	pos	13.41	165.05	Level 2	Endogenous	aminoacids and derivatives	No								
creatinine	pos	1.31	227.13	Level 2	Endogenous	aminoacids and derivatives	No								
ursodeoxycholic acid	neg	16.18	805.56	Level 2	Endogenous	bile acids	No								
Cholic Acid_2	neg	15.15	815.57	Level 2	Endogenous	bile acids	No								
glycodeoxycholic acid_1	neg	14.80	919.60	Level 2	Endogenous	bile acids	No								
3-oxo-5beta-cholanic acid	pos	14.39	375.29	Level 2	Endogenous	bile acids	No								
deoxycholic acid	pos	13.59	375.29	Level 2	Endogenous	bile acids	No								
Tauroursodeoxycholic acid_2	pos	14.84	500.30	Level 2	Endogenous	bile acids	No								
Cholic Acid_4	pos	15.16	834.61	Level 2	Endogenous	bile acids	No								
glycolithocholic acid_3	pos	13.38	867.65	Level 2	Endogenous	bile acids	No								
glycohyodeoxycholic acid	pos	13.24	899.64	Level 2	Endogenous	bile acids	No								
Heptadecanoic acid	neg	18.65	269.25	Level 2	Endogenous	fatty acids and derivatives	No								
16-Hydroxyhexadecanoic acid	neg	15.81	271.23	Level 2	Endogenous	fatty acids and derivatives	No								
10-Hydroxydecanoic acid	pos	18.05	171.14	Level 2	Endogenous	fatty acids and derivatives	No								
12-Hydroxydodecanoic acid	pos	18.57	217.18	Level 2	Endogenous	fatty acids and derivatives	No								
cis-9-Hexadecenoic acid	pos	15.79	237.22	Level 2	Endogenous	fatty acids and derivatives	No								
Palmitelaidic acid	pos	17.79	237.22	Level 2	Endogenous	fatty acids and derivatives	No								
gamma-linolenic acid	pos	15.12	279.23	Level 2	Endogenous	fatty acids and derivatives	No								
Linoleic acid_6	pos	17.10	279.23	Level 2	Endogenous	fatty acids and derivatives	No								
Linoleic acid_3	pos	18.41	281.25	Level 2	Endogenous	fatty acids and derivatives	No								
Pentadecanoyl Ethanolamide	pos	18.33	286.27	Level 2	Endogenous	fatty acids and derivatives	No								
15-HEDE	pos	16.89	307.26	Level 2	Endogenous	fatty acids and derivatives	No								
9Z,11E,13E-Octadecatrienoic acid ethyl ester	pos	13.70	307.26	Level 2	Endogenous	fatty acids and derivatives	No								
8-hydroxy-8-(3-octyloxiran-2-yl)octanoic acid	pos	18.36	315.25	Level 2	Endogenous	fatty acids and derivatives	No								
9S,11R-Epidioxy-15S-hydroxy-13E-prostaenoic acid	pos	16.14	337.23	Level 2	Endogenous	fatty acids and derivatives	No								
Stearoyl-carnitine	pos	18.39	428.37	Level 2	Endogenous	fatty acids and derivatives	No								
Levothyroxine	neg	13.73	775.68	Level 2	Endogenous	hormones and metabolites	No								
androstene-3a-17b-diol	pos	17.45	273.22	Level 2	Endogenous	hormones and metabolites	No								
17-Methyltestosterone_1	pos	16.61	303.23	Level 2	Endogenous	hormones and metabolites	No								
6-beta-Hydroxycortisol	pos	14.48	379.21	Level 2	Endogenous	hormones and metabolites	No								
4-hydroxybenzoate	neg	5.18	137.02	Level 2	Endogenous	other endogenous metabolites	No								
3-methylsalicylic acid	neg	11.21	151.04	Level 2	Endogenous	other endogenous metabolites	No								
2-Hydroxyphenylacetic acid	neg	7.03	151.04	Level 2	Endogenous	other endogenous metabolites	No								
mandelic acid	neg	12.68	151.04	Level 2	Endogenous	other endogenous metabolites	No								
3-hydroxybenzyl alcohol	pos	10.30	107.05	Level 2	Endogenous	other endogenous metabolites	No								

4-methoxy-3-methylbenzaldehyde	pos	14.89	133.06	Level 2	Endogenous	other endogenous metabolites	No
Pyridoxal	pos	9.48	168.07	Level 2	Endogenous	other endogenous metabolites	No
Vitamin K1	pos	12.40	226.18	Level 2	Endogenous	other endogenous metabolites	No
1-Methyladenosine	pos	6.29	282.12	Level 2	Endogenous	other endogenous metabolites	No
Retinoic acid	pos	19.70	301.22	Level 2	Endogenous	other endogenous metabolites	No
alpha-Tocopherol	pos	20.88	430.38	Level 2	Endogenous	other endogenous metabolites	No
Hemin cation	pos	16.56	616.18	Level 2	Endogenous	other endogenous metabolites	No
PI 36	neg	19.11	861.55	Level 2	Endogenous	sterol and phospholipids and derivatives	No
1-Octadecanoyl-2-(5Z,8Z,11Z,14Z-eicosatetraenoyl)-sn-glycero-3-phospho-(1'-myo-inositol)	neg	19.16	885.55	Level 2	Endogenous	sterol and phospholipids and derivatives	No
desmosterol	pos	21.31	367.34	Level 2	Endogenous	sterol and phospholipids and derivatives	No
Cholesterol	pos	21.34	369.35	Level 2	Endogenous	sterol and phospholipids and derivatives	No
1-Palmitoyl-2-linoleoyl-sn-glycero-3-phosphocholine	pos	21.22	758.57	Level 2	Endogenous	sterol and phospholipids and derivatives	No
1-Hexadecanoyl-2-(9Z-octadecenoyl)-sn-glycero-3-phosphocholine	pos	20.94	782.57	Level 2	Endogenous	sterol and phospholipids and derivatives	No
1-Palmitoyl-2-docosahexaenoyl-sn-glycero-3-phosphocholine	pos	20.90	806.57	Level 2	Endogenous	sterol and phospholipids and derivatives	No
Carbamazepine	pos	11.97	237.10	Level 1	Environmental	drugs and metabolites	No
atenolol	pos	5.12	267.17	Level 1	Environmental	drugs and metabolites	No
Fluconazole	pos	9.00	307.11	Level 1	Environmental	drugs and metabolites	No
2-Chloro-4,6-dinitroaniline	neg	11.78	215.98	Level 1	Environmental	industrial chemicals and metabolites	No
Salicylate	neg	10.94	137.02	Level 2	Environmental	drugs and metabolites	No
4-(Trifluoromethyl)phenol	neg	13.56	161.02	Level 2	Environmental	drugs and metabolites	No
4-Trifluoromethylphenol	neg	14.92	161.02	Level 2	Environmental	drugs and metabolites	No
Gemfibrozil	neg	15.87	249.15	Level 2	Environmental	drugs and metabolites	No
Letrozole	neg	10.92	284.10	Level 2	Environmental	drugs and metabolites	No
Hydrochlorothiazide	neg	4.54	295.96	Level 2	Environmental	drugs and metabolites	No
Warfarin	neg	12.00	307.10	Level 2	Environmental	drugs and metabolites	No
4`-Hydroxy Diclofenac	neg	11.45	310.00	Level 2	Environmental	drugs and metabolites	No
Losartan	neg	12.40	421.15	Level 2	Environmental	drugs and metabolites	No
Atorvastatin	neg	13.80	557.25	Level 2	Environmental	drugs and metabolites	No
Isodeoxycholic acid	neg	15.04	783.58	Level 2	Environmental	drugs and metabolites	No
Cathine	pos	5.49	152.11	Level 2	Environmental	drugs and metabolites	No
Gaultherin	pos	11.92	153.05	Level 2	Environmental	drugs and metabolites	No
Gabapentin	pos	11.15	172.13	Level 2	Environmental	drugs and metabolites	No
Memantine	pos	13.50	180.17	Level 2	Environmental	drugs and metabolites	No
Aspirin	pos	13.86	181.05	Level 2	Environmental	drugs and metabolites	No
Iminostilbene	pos	11.72	194.10	Level 2	Environmental	drugs and metabolites	No
N6-benzyl-7H-purine-2,6-diamine	pos	16.50	241.12	Level 2	Environmental	drugs and metabolites	No
Carbamazepine-10,11-epoxide	pos	10.38	253.10	Level 2	Environmental	drugs and metabolites	No
Ketoprofen_1	pos	10.81	255.10	Level 2	Environmental	drugs and metabolites	No
Ketoprofen_2	pos	8.67	255.10	Level 2	Environmental	drugs and metabolites	No
Ketoprofen_3	pos	11.64	255.10	Level 2	Environmental	drugs and metabolites	No
Mianserin	pos	14.40	265.17	Level 2	Environmental	drugs and metabolites	No
Atenolol acid	pos	5.43	268.15	Level 2	Environmental	drugs and metabolites	No
Mianserin-N-Oxide	pos	12.60	281.17	Level 2	Environmental	drugs and metabolites	No
Nitrazepam	pos	12.24	282.09	Level 2	Environmental	drugs and metabolites	No
metenolone	pos	19.72	285.22	Level 2	Environmental	drugs and metabolites	No
Oxazepam	pos	12.92	287.06	Level 2	Environmental	drugs and metabolites	No
h-61-17-epioxandrolone	pos	16.07	289.22	Level 2	Environmental	drugs and metabolites	No
Trimethoprim	pos	7.39	291.15	Level 2	Environmental	drugs and metabolites	No
Sertraline	pos	14.02	306.08	Level 2	Environmental	drugs and metabolites	No
Rofecoxib	pos	10.84	315.07	Level 2	Environmental	drugs and metabolites	No
Citalopram	pos	11.30	325.17	Level 2	Environmental	drugs and metabolites	No
Norpropoxyphene_1	pos	14.83	326.21	Level 2	Environmental	drugs and metabolites	No
Norpropoxyphene_2	pos	12.84	326.21	Level 2	Environmental	drugs and metabolites	No
Propoxyphene	pos	13.16	340.23	Level 2	Environmental	drugs and metabolites	No
Canrenone	pos	15.68	341.21	Level 2	Environmental	drugs and metabolites	No
3Beta-Hydroxy-23,24-Bisnorchol-5-Enic Acid	pos	16.92	369.24	Level 2	Environmental	drugs and metabolites	No
Bufogenin	pos	9.91	385.23	Level 2	Environmental	drugs and metabolites	No
Dixyrazine	pos	14.98	428.24	Level 2	Environmental	drugs and metabolites	No
Verapamil	pos	10.75	455.29	Level 2	Environmental	drugs and metabolites	No
6-methoxypurine	neg	8.28	149.05	Level 2	Environmental	foods and additives	No
Umbelliferone	neg	9.59	161.02	Level 2	Environmental	foods and additives	No
Isoeugenol	neg	9.60	163.08	Level 2	Environmental	foods and additives	No
Naringenin	neg	11.53	271.06	Level 2	Environmental	foods and additives	No
Chlorogenic acid	neg	4.34	353.09	Level 2	Environmental	foods and additives	No
Hydroferulic acid	neg	7.06	391.14	Level 2	Environmental	foods and additives	No
Ursolic Acid	neg	18.14	455.35	Level 2	Environmental	foods and additives	No
Tiliroside	neg	11.26	593.13	Level 2	Environmental	foods and additives	No
4-aminophenol	pos	4.73	110.06	Level 2	Environmental	foods and additives	No
carveol	pos	8.79	135.12	Level 2	Environmental	foods and additives	No
Coumarin	pos	9.54	147.04	Level 2	Environmental	foods and additives	No
Methyl trans-styryl ketone	pos	10.35	147.08	Level 2	Environmental	foods and additives	No
myrcene_2	pos	11.24	159.12	Level 2	Environmental	foods and additives	No
4-Hydroxycoumarin_2	pos	16.12	163.04	Level 2	Environmental	foods and additives	No
4-Hydroxycoumarin_4	pos	10.86	163.04	Level 2	Environmental	foods and additives	No
3-Methoxycinnamic acid	pos	17.79	179.07	Level 2	Environmental	foods and additives	No

gamma-Dodecalactone	pos	18.46	199.17	Level 2	Environmental	foods and additives	No
Pterosin B	pos	12.02	219.14	Level 2	Environmental	foods and additives	No
Isokobusone	pos	17.75	223.17	Level 2	Environmental	foods and additives	No
senkyunolide	pos	11.15	225.11	Level 2	Environmental	foods and additives	No
N-Benzyladenine	pos	10.13	226.11	Level 2	Environmental	foods and additives	No
(+)-Costunolide	pos	15.83	233.15	Level 2	Environmental	foods and additives	No
Pterosin A	pos	11.30	249.15	Level 2	Environmental	foods and additives	No
Sclareolide	pos	18.43	251.20	Level 2	Environmental	foods and additives	No
elaidic acid	pos	19.08	283.26	Level 2	Environmental	foods and additives	No
6-Gingerol	pos	13.38	295.19	Level 2	Environmental	foods and additives	No
Conjugated linoleic Acid (10E,12Z)	pos	18.16	299.26	Level 2	Environmental	foods and additives	No
(E)-5-(1,2,4a,5-tetramethyl-7-oxo-3,4,8,8a-tetrahydro-2H-naphthalen-1-yl)-3-methylpent-2-enoic acid	pos	17.49	319.23	Level 2	Environmental	foods and additives	No
Bisdemethoxycurcumin	pos	7.62	331.09	Level 2	Environmental	foods and additives	No
5-[5-hydroxy-3-(hydroxymethyl)pentyl]-8a-(hydroxymethyl)-5,6-dimethyl-3,4,4a,6,7,8-hexahydronaphthalene-1-carboxylic acid	pos	13.64	337.24	Level 2	Environmental	foods and additives	No
quinine	pos	11.05	347.17	Level 2	Environmental	foods and additives	No
2-[4a-methyl-8-methylidene-4-(3-methylpentanoyloxy)-1,2,3,4,5,6,7,8a-octahydronaphthalen-2-yl]prop-2-enoic acid	pos	14.57	366.26	Level 2	Environmental	foods and additives	No
Digitoxigenin_2	pos	9.56	375.25	Level 2	Environmental	foods and additives	No
Methyl rosmarinate	pos	7.71	397.09	Level 2	Environmental	foods and additives	No
Demissidine	pos	14.97	400.36	Level 2	Environmental	foods and additives	No
enoxolone	pos	16.90	453.34	Level 2	Environmental	foods and additives	No
m-xylene-4-sulfonic acid	neg	12.49	185.03	Level 2	Environmental	industrial chemicals and metabolites	No
Acridone	neg	11.81	194.06	Level 2	Environmental	industrial chemicals and metabolites	No
2,6-Di-tert-butyl-4-nitrophenol	neg	16.33	250.15	Level 2	Environmental	industrial chemicals and metabolites	No
o-Toluidine	pos	11.40	108.08	Level 2	Environmental	industrial chemicals and metabolites	No
1-Methyl-1,2,3-benzotriazole	pos	9.68	134.07	Level 2	Environmental	industrial chemicals and metabolites	No
Diethylene glycol monobutyl ether	pos	10.38	163.13	Level 2	Environmental	industrial chemicals and metabolites	No
Phthalic acid	pos	6.59	167.03	Level 2	Environmental	industrial chemicals and metabolites	No
2-Mercaptobenzothiazole	pos	12.38	167.99	Level 2	Environmental	industrial chemicals and metabolites	No
1-Cyclohexylpyrrolidin-2-one	pos	11.28	168.14	Level 2	Environmental	industrial chemicals and metabolites	No
Dicyclohexylamine	pos	9.49	182.19	Level 2	Environmental	industrial chemicals and metabolites	No
Triethyl phosphate	pos	10.41	183.08	Level 2	Environmental	industrial chemicals and metabolites	No
Dibenzylamine	pos	9.45	198.13	Level 2	Environmental	industrial chemicals and metabolites	No
1,3-Diphenylguanidine	pos	8.15	212.12	Level 2	Environmental	industrial chemicals and metabolites	No
2-Isopropylthioxanthone	pos	16.94	255.08	Level 2	Environmental	industrial chemicals and metabolites	No
Iodopropynyl butylcarbamate (IPBC)	pos	12.59	282.00	Level 2	Environmental	industrial chemicals and metabolites	No
Denatonium	pos	11.17	325.23	Level 2	Environmental	industrial chemicals and metabolites	No
p-tert-Octylphenol pentaglycol ether	pos	17.38	427.31	Level 2	Environmental	industrial chemicals and metabolites	No
3-Ethoxy-4-hydroxybenzaldehyde	neg	11.39	165.05	Level 2	Environmental	other contaminants and metabolites	No
2-Amino-9H-pyrido[2,3-b]indole (AalphaC)	pos	5.89	184.09	Level 2	Environmental	other contaminants and metabolites	No
Coumatetralyl	pos	10.14	293.12	Level 2	Environmental	other contaminants and metabolites	No
Ectoine	neg	10.77	141.00	Level 2	Environmental	personal care chemicals and metabolites	No
Phenylparaben	neg	13.19	213.06	Level 2	Environmental	personal care chemicals and metabolites	No
Hexylparaben	neg	11.77	221.12	Level 2	Environmental	personal care chemicals and metabolites	No
N,N-Dimethyldodecylamine N-oxide	pos	16.22	230.25	Level 2	Environmental	personal care chemicals and metabolites	No
N-[3-(Dimethylamino)propyl]dodecanamide	pos	15.78	285.29	Level 2	Environmental	personal care chemicals and metabolites	No
Lauryl diethanolamide	pos	16.11	288.25	Level 2	Environmental	personal care chemicals and metabolites	No
Myristamidopropyl betaine	pos	19.12	371.33	Level 2	Environmental	personal care chemicals and metabolites	No
Didodecyl 3,3'-thiodipropionate oxide_1	pos	20.87	531.41	Level 2	Environmental	personal care chemicals and metabolites	No
Didodecyl 3,3'-thiodipropionate oxide_2	pos	20.09	553.39	Level 2	Environmental	personal care chemicals and metabolites	No
2-Phenylphenol_2	pos	9.32	171.08	Level 2	Environmental	pesticides and metabolites	No
2-Phenylphenol_1	pos	7.86	171.08	Level 2	Environmental	pesticides and metabolites	No
Atrazine-desisopropyl	pos	12.79	174.05	Level 2	Environmental	pesticides and metabolites	No
aldicarb	pos	10.96	191.09	Level 2	Environmental	pesticides and metabolites	No
2-Aminosulfonyl-benzoic acid methyl ester	pos	14.49	216.03	Level 2	Environmental	pesticides and metabolites	No
Thiamethoxam	pos	6.94	292.03	Level 2	Environmental	pesticides and metabolites	No
Diazinon	pos	15.39	305.11	Level 2	Environmental	pesticides and metabolites	No
Bioresmethrin	pos	16.49	339.20	Level 2	Environmental	pesticides and metabolites	No
(4S,5Z,6S)-4-(2-methoxy-2-oxoethyl)-5-[2-[(E)-3-phenylprop-2-enoyl]oxyethylidene]-6-[(2S,3R,4S,5S,6R)-3,4,5-trihydroxy-6-	neg	14.15	247.13	Level 2	Environmental	plant metabolites and natural products	No
Rhyncophylline	neg	6.44	383.19	Level 2	Environmental	plant metabolites and natural products	No
Paeonol_1	pos	8.40	167.07	Level 2	Environmental	plant metabolites and natural products	No
Paeonol_2	pos	7.15	167.07	Level 2	Environmental	plant metabolites and natural products	No
Lupinine	pos	9.95	170.15	Level 2	Environmental	plant metabolites and natural products	No
2-Butanone, 4-(2,6,6-trimethyl-2-cyclohexen-1-yl)-	pos	20.02	177.16	Level 2	Environmental	plant metabolites and natural products	No
Pestalotin	pos	9.17	215.13	Level 2	Environmental	plant metabolites and natural products	No
Isoalantolactone	pos	12.82	215.14	Level 2	Environmental	plant metabolites and natural products	No
Thujopsenone	pos	12.86	219.17	Level 2	Environmental	plant metabolites and natural products	No
alpha-Cyperone	pos	16.78	219.17	Level 2	Environmental	plant metabolites and natural products	No
Manool	pos	18.56	273.26	Level 2	Environmental	plant metabolites and natural products	No
methylidenetetracyclo[11.2.1.0??,?????.0???,???]hexadec-10-ene-5-carboxylic acid	pos	16.76	301.22	Level 2	Environmental	plant metabolites and natural products	No
1,4a-dimethyl-6-methylidene-5-[(2E)-3-methylpenta-2,4-dienyl]-3,4,5,7,8,8a-hexahydro-2H-naphthalene-1-carboxylic acid	pos	18.24	303.23	Level 2	Environmental	plant metabolites and natural products	No

Methylophiopogonanone B	pos	13.29	329.14	Level 2	Environmental	plant metabolites and natural products	No
16,17-Dihydroxykauran-18-oic acid	pos	12.72	354.26	Level 2	Environmental	plant metabolites and natural products	No
Deoxyelephantopin	pos	14.99	367.11	Level 2	Environmental	plant metabolites and natural products	No
Eleutheroside B	pos	10.35	373.15	Level 2	Environmental	plant metabolites and natural products	No
Ulbactin F	pos	14.68	394.07	Level 2	Environmental	plant metabolites and natural products	No
Zizyberanalic acid	pos	16.56	488.37	Level 2	Environmental	plant metabolites and natural products	No
alpha-Chaconine	pos	13.15	852.51	Level 2	Environmental	plant metabolites and natural products	No
Dibutyl phthalate	pos	14.11	279.16	Level 2	Environmental	plasticizers and metabolites	No
Neopentyl glycol dibenzoate	pos	16.04	313.14	Level 2	Environmental	plasticizers and metabolites	No
Diethylene glycol dibenzoate	pos	14.61	315.12	Level 2	Environmental	plasticizers and metabolites	No
Triethylene glycol dibenzoate	pos	12.12	359.15	Level 2	Environmental	plasticizers and metabolites	No
